# Dramatic Increase in Content of Diverse Flavonoids Accompanied with Down-Regulation of F-Box Genes in a Chrysanthemum (*Chrysanthemum* × *morifolium* (Ramat.) Hemsl.) Mutant Cultivar Producing Dark-Purple Ray Florets

**DOI:** 10.3390/genes11080865

**Published:** 2020-07-30

**Authors:** Yeong Deuk Jo, Jaihyunk Ryu, Ye-Sol Kim, Kyung-Yun Kang, Min Jeong Hong, Hong-Il Choi, Gah-Hyun Lim, Jin-Baek Kim, Sang Hoon Kim

**Affiliations:** 1 Radiation Breeding Research Team, Advanced Radiation Technology Institute, Korea Atomic Energy Research Institute, Jeongeup 56212, Korea; jyd@kaeri.re.kr (Y.D.J.); jhryu@kaeri.re.kr (J.R.); jollysoul2@gmail.com (Y.-S.K.); hongmj@kaeri.re.kr (M.J.H.); hichoi@kaeri.re.kr (H.-I.C.); kah7702@kaeri.re.kr (G.-H.L.); jbkim74@kaeri.re.kr (J.-B.K.); 2 Suncheon Research Center for Natural Medicines, Suncheon 57922, Korea; nms-kang@nate.com

**Keywords:** flavonoid, anthocyanin, F-box protein, mutation, chrysanthemum

## Abstract

Anthocyanins (a subclass of flavonoids) and flavonoids are crucial determinants of flower color and substances of pharmacological efficacy, respectively, in chrysanthemum. However, metabolic and transcriptomic profiling regarding flavonoid accumulation has not been performed simultaneously, thus the understanding of mechanisms gained has been limited. We performed HPLC-DAD-ESI-MS (high-performance liquid chromatography coupled with photodiode array detection and electrospray ionization mass spectrometry) and transcriptome analyses using “ARTI-Dark Chocolate” (AD), which is a chrysanthemum mutant cultivar producing dark-purple ray florets, and the parental cultivar “Noble Wine” for metabolic characterization and elucidation of the genetic mechanism determining flavonoid content. Among 26 phenolic compounds identified, three cyanidins and eight other flavonoids were detected only in AD. The total amounts of diverse flavonoids were 8.0 to 10.3 times higher in AD. Transcriptome analysis showed that genes in the flavonoid biosynthetic pathway were not up-regulated in AD at the early flower stage, implying that the transcriptional regulation of the pathway did not cause flavonoid accumulation. However, genes encoding post-translational regulation-related proteins, especially F-box genes in the mutated gene, were enriched among down-regulated genes in AD. From the combination of metabolic and transcriptomic data, we suggest that the suppression of post-translational regulation is a possible mechanism for flavonoid accumulation in AD. These results will contribute to research on the regulation and manipulation of flavonoid biosynthesis in chrysanthemum.

## 1. Introduction

The flavonoids are a class of phenylpropanoids with a C6-C3-C6 carbon skeleton comprising more than 10,000 structures [1]. Not only owing to their importance in plant biological functions, such as protection from ultraviolet radiation [2] and defense against pathogens and herbivores [3], flavonoids have received substantial attention for a wide variety of human uses as antioxidants or colorants for pharmaceutical products, cosmetics, and food products [4]. In addition, a subclass of flavonoids known as anthocyanins are important natural pigments responsible for the characteristic colors in flowers and fruits [5].

Flavonoids are synthesized by means of the phenylpropanoid pathway, which is highly conserved among plant taxa. In this pathway, which is initiated with the metabolism of phenylalanine, chalcone synthase (CHS) and chalcone isomerase (CHI) catalyze the synthesis of the central intermediate of flavonoids, naringenin [4]. The well-known pigments, anthocyanins, are synthesized by subsequent reactions by enzymes coded by structural genes of the anthocyanin biosynthesis pathway. In addition, diverse types of other flavonoids, including flavonols, flavanones, and flavans, are generated from reactions catalyzed by other enzymes using intermediates of the anthocyanin biosynthesis pathway as substrates [6].

The enzymes involved in flavonoid biosynthesis or transport are regulated by mechanisms at a variety of genetic levels. At the transcription level, early flavonoid biosynthetic genes such as *CHS*, *CHI*, -flavanone 3-hydroxylase (*F3H*), and flavonol synthase (*FLS*) are regulated by R2R3-MYB proteins [6]. In addition, the MYB-bHLH-WD40 (MBW) complex, composed of an R2R3-MYB protein, a protein with a basic helix-loop-helix (bHLH) domain, and a WD40 protein, is an important activator for late flavonoid biosynthetic genes and genes of transporters that are involved in the import of flavonoids into the vacuole [7,8]. In contrast, several MYB transcription factors act as negative regulators by inhibiting the expression of positive regulators [9] or the formation of the MBW complex [10]. Post-transcriptional regulation is also involved in the control of these transcription factors, as illustrated by the negative regulation of PAP1, PAP2, and MYB113 (R2R3-MYB transcription factors of *Arabidopsis*) by the small interfering RNA *TAS2*-siR81(−) [11] and of SPL9 by a microRNA, miR156, which leads to the destabilization of the MBW complex in *Arabidopsis* [12].

More recently, post-translational regulation has been reported [13,14,15]. In post-translational regulation involving ubiquitination, the specificity of the target protein is conferred by selective interactions between target proteins and E3 ubiquitin ligases [16]. Zhang et al. [14,15] showed that four Kelch motif-containing F-box (KFB) proteins, which are components of a class of E3 ligases, physically interact with phenylalanine ammonia-lyase (PAL), leading to the ubiquitination of PAL and, ultimately, the degradation of PAL in *Arabidopsis*. Given that PAL catalyzes the first and committed reaction in the phenylpropanoid pathway, mutation of the four F-box proteins results in the accumulation of a variety of flavonoids and lignins. In addition, a KFB protein of *Arabidopsis* interacts with CHS, and, in turn, results in the negative regulation of flavonoid biosynthesis in response to exogenous stimuli [13]. In contrast, an increase in the sumoylation of R2R3-MYB and MdMYB1 interrupts their ubiquitination, thus leading to the up-regulation of anthocyanin biosynthesis genes in apple [17].

Chrysanthemum ranks second in terms of the international trade of flowers after roses, accounting for more than 30% of world cut flower production [18]. In chrysanthemum, anthocyanins (a subclass of flavonoids) are crucial determinants of flower color, together with carotenoids [19]. Therefore, flavonoid identity and the mechanisms regulating their production have been the focus of many studies. For example, flavonoid contents were screened by metabolite analysis [20,21]. In addition, genetic regulators in flavonoid biosynthesis, according to environmental cues or developmental stages, were investigated by transcriptome analysis [22,23]. Finally, the manipulation of flower color by the engineering of anthocyanin content has been attempted by means of transformation [24,25] and artificial mutagenesis [26,27]. However, no integrative study including metabolome and transcriptome analyses has been conducted to understand the mechanism underlying the artificial modification of flavonoid content in chrysanthemum.

We have developed a series of chrysanthemum mutant cultivars by γ-irradiation on “Noble Wine”, which is a spray-type Korean commercial cultivar with bright stripes on multiple flowers. One such mutant cultivar, “ARTI-Yellow Star”, has yellow ray florets and was revealed to highly accumulate carotenoids [28]. In this cultivar, the deletion of *CmCCD4a* genes that have been reported to degrade carotenoids was detected [28]. Another mutant cultivar, “ARTI-Dark Chocolate”, which produces dark purple ray florets, was revealed to accumulate a large amount of flavonoids and have strong antioxidant capacity [29]. However, metabolic analysis has not been performed specifically on the ray floret corolla, and a genomics study to elucidate the mechanism for flavonoid accumulation has not been attempted in this cultivar.

In this study, we performed high-performance liquid chromatography coupled with photodiode array detection and electrospray ionization mass spectrometry (HPLC-DAD-ESI-MS) and transcriptome analysis using an artificially mutated chrysanthemum cultivar that produces dark purple ray florets to characterize flavonoid content in the ray floret corolla and to elucidate the mechanism of flavonoid accumulation.

## 2. Materials and Methods

### 2.1. Plant Materials and Analysis of Flower Color

**Figure 1:**
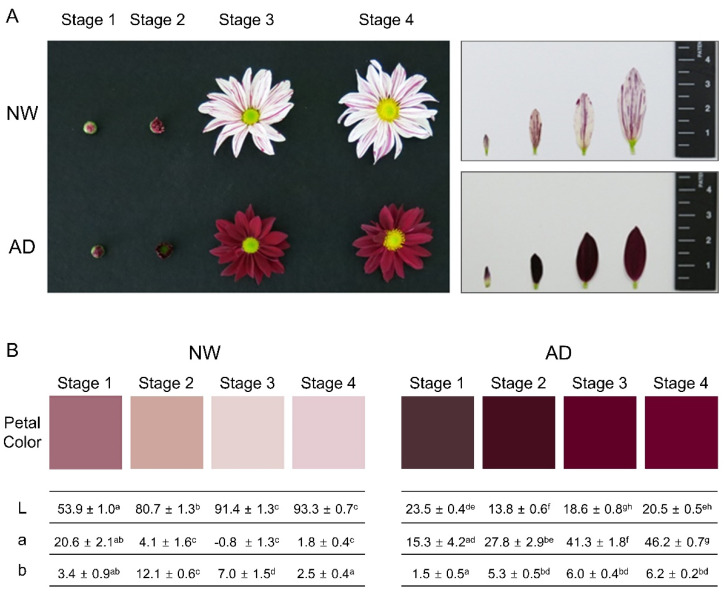
Analysis of ray floret corolla color at four capitulum developmental stages in “Noble Wine” and “ARTI-Dark Chocolate”. (**A**) Capitulum morphology and corolla color at each stage. (**B**) Color coordinates in lab color space for ray floret corolla color at each stage. Color coordinates were measured at each stage in three individuals using a colorimeter. Values are the mean ± standard error (*n* = 3). L, a, and b indicate lightness from black (0) to white (100), color from green (-) to red (+), and color from blue (-) to yellow (+), respectively. Different letters to the right of L, a, and b values indicate statistical differences analyzed by one-way ANOVA and Duncan’s multiple range test.

“ARTI-Dark Chocolate” is a chrysanthemum (*Chrysanthemum* × *morifolium* (Ramat.) Hemsl.) cultivar with dark purple ray florets that was selected from γ-irradiated (50 Gy) stem cuttings of the cultivar “Noble Wine” (Figure 1A). Both cultivars were cultivated without artificial light control in a glasshouse at the Advanced Radiation Technology Institute (Jeongeup, Jeollabuk-do, Korea) repeatedly in 2014 and 2019. The plants cultivated in 2014 were used for metabolic, transcriptomic, and qRT-PCR analyses, while those cultivated in 2019 were used only for qRT-PCR analysis to test the reproducibility of the gene expression analyses in which samples obtained in 2014 were used. During the flower development stages in the two cultivation periods, the temperature was maintained between 17 and 22 °C, and the light intensity was around 12,000–17,000 l× at noon. Capitulum development was classified into four stages in accordance with Jo et al. [28]. At stage 1, the involucral bracts were partially open and the upper portion of the ray floret corolla was visible; at stage 2, the ray floret corolla was exserted and partially unfurled; at stages 3 and 4, the ray floret corolla was fully expanded and the disc florets were either all closed or at least some were open, respectively (Figure 1B). We performed HPLC-DAD-ESI-MS analysis of the ray floret corollas of the two cultivars (cultivated in 2014) at all four stages with three experimental replications. In addition, a transcriptome analysis was performed for ray floret corollas at stage 1. In transcriptome analysis, ray floret corollas pooled from five individuals of each cultivar were used as material. The qRT-PCR analyses were performed for three individuals of each cultivar. The color of the ray floret corollas from three individuals of each cultivar was analyzed using a Chroma Meter CR-400 (Monica Minolta, Inc., Tokyo, Japan). Differences in L, a, and b values between cultivars and stages were statistically analyzed by one-way ANOVA and Duncan’s multiple rage test using IBM SPSS Statistics v22.0 (IBM, Amonk, NY, USA).

### 2.2. Extraction of Phenolic Compounds

The procedure for the extraction of phenolic compounds followed that of Chen et al. [30]. Briefly, 0.5 g freeze-dried ray floret corollas were extracted in 3 mL of methanol:water:formic acid:TFA (70:27:2:1, *v*/*v*) solution at 4 °C in the dark for 24 h, with vortexing every 6 h. The solution was filtered using a 0.2 μm membrane filter. Three replicate extractions per sample were conducted.

### 2.3. HPLC-DAD-ESI-MS Analysis

Each sample extract was analyzed using an HPLC and a photodiode array detector (DAD; Agilent 1260 series; Agilent Technologies, Santa Clara, CA, USA) and quadrupole liquid chromatograph/mass spectrometer (Agilent 6130; Agilent Technologies, Santa Clara, CA, USA) equipped with a Poroshell 120 SB-C18 column (150 × 4.6 mm i.d., 2.7 m particle size; Agilent Technologies) and a compatible C18 guard column (4 × 3 mm i.d.; 3 μm particle size; Phenomenex, Torrance, CA, USA). The mobile phase was composed of water (A, 0.05% formic acid) and acetonitrile (B, 0.05% formic acid). The gradient program was 0–3 min, 95% A and 5% B; 3–28 min, 100% B; and 28–35 min, 100% B. The flow rate of the mobile phase was adjusted to 0.5 mL/min and the column temperature was set to 24 ℃. The injection volume was 10 µL. The optimal atmospheric pressure ionization–electrospray ionization parameters were determined as 12.0 L/min drying gas pressure, 3000 V capillary voltage positive and negative, 350 °C drying gas temperature, and 35 psig nebulizer gas pressure. The mass selective detector signal set with positive and negative ionization were fragmentor 70, mass range 100–1000, and scan mode. Quercetin and cyanidin were used as standards for the quantification of extracts in analysis at 350 and 515 nm, respectively. All aforementioned chemicals and the standards were of analytical grade and purchased from Sigma-Aldrich (Shanghai, China). Differences in the amounts of metabolites between cultivars and stages were statistically analyzed by one-way ANOVA and Duncan’s multiple rage test using IBM SPSS Statistics v22.0 (IBM, Amonk, NY, USA).

### 2.4. Transcriptome Analysis

Transcriptome analysis was performed following the method described by Jo et al. [28]. Briefly, a cDNA library was constructed using total RNA extracted from the ray floret corollas of the two cultivars at stage 1. Sequencing of the cDNA libraries was performed using an Illumina HiSeq2000 Sequencing System (Illumina, San Diego, CA, USA) at the Theragen Etex Bio Institute (Suwon, Korea). After the elimination of low-quality reads (Q score < 20 in average or in 40% of the bases), the ends of reads were trimmed if the associated quality scores were less than 20. The assembly, grouping, and extraction of representative sequences were performed using Trinity [31,32], TGICL [33], and CAP3 [34], respectively. The transcriptome sequencing reads generated from NW and AD have been deposited in the Sequence Read Archive (SRA) database of National Center for Biotechnology Information (NCBI; BioProject ID: PRJNA633115).

### 2.5. Annotation, Functional Analysis, and Expression Level Determination of Unigenes

From the finally assembled sequences that were considered to be unigenes, protein coding sequences (CDSs) were extracted using TransDecoder [33], and annotated using BLAST (version 2.2.28+; E-value < 1 × 10^−5^) and InterProScan (version 5; E-value < 1 × 10^−5^). Gene ontology (GO) analysis was performed based on Fisher’s exact test (*p*-value < 0.001). Expression levels of the unigenes were analyzed using RSEM (RNA-Seq by Expectation Maximization), which quantifies the expression of transcripts without reference information [35].

### 2.6. Analysis of Differentially Expressed Genes

The analysis of differentially expressed genes (DEGs), applying the iterative DEGEN/DEseq method, was performed using the TCC package [36]. The normalization to screen meaningful DEGs was performed three times [37]. The *p*-values were calculated based on DEseq by using a negative binomial distribution [36]. The DEGs were determined based on the *p*-value threshold of 0.005. The DEGs were classified according to level 2 GO terms using the WEGO tool [38]. Annotation based on information in the Kyoto Encyclopedia of Genes and Genomes (KEGG) and euKaryotic Orthologous Groups (KOG) databases was performed using the KEGG Automatic Annotation Server [39] and the Web Services for Metagenomics Analysis server [40]. Gene functional clustering and enrichment analysis were performed using the DAVID functional annotation tool (version 6.7) [41], in which UniProt IDs assigned to unigenes by the InterProScan tool were used as identifiers.

### 2.7. Quantitative Reverse Transcription PCR and Unigene PCR Amplification

We performed quantitative reverse transcription PCR (qRT-PCR) analysis using RNAs extracted from ray floret corollas of NW and AD plants. This analysis was performed repeatedly using samples from plants cultivated in 2014 and 2019, respectively. The first-strand cDNA was synthesized from 1 µg total RNA using M-MLV Reverse Transcriptase (Promega, Madison, WI, USA). Quantitative reverse transcription PCR (qRT-PCR) analyses were performed using SYBR Premix Ex Taq II (Takara Bio, Otsu, Japan) and the CFX96 Touch Real-Time PCR Detection System (Bio-Rad, Hercules, CA, USA). The selected unigenes were amplified by PCR from 50 ng genomic DNA with 32 cycles. The sequences of the U0233278-specific primers were 5′-GTTCAAAATACTACCCGATCCTGAC-3′ and 5′-GAATGGCCCCATTGCACAAG-3′. The correlation between transcriptome and qRT-PCR analyses was evaluated by calculating the Pearson correlation coefficient (*r*) and *p*-value using IBM SPSS Statistics v22.0 (IBM, Amonk, NY, USA). The differences in expression levels between cultivars and stages were statistically analyzed by one-way ANOVA and Duncan’s multiple rage test using IBM SPSS Statistics v22.0 (IBM, Amonk, NY, USA).

## 3. Results

### 3.1. Analysis of Ray Floret Corolla Color of “Noble Wine” and “ARTI-Dark Chocolate”

“ARTI-Dark Chocolate” is a mutant cultivar producing dark purple ray florets developed by γ-irradiation on an original cultivar, “Noble Wine”, that produces very light pink ray florets with purple-colored strips (Figure 1A; hereafter, NW and AD will be used as abbreviations for Noble Wine and ARTI-Dark Chocolate, respectively). The ray floret corolla colors of AD and NW were comparatively analyzed based on the lab color model at four capitulum developmental stages (Figure 1B). The L value was considerably lower in AD than in NW at all stages (a low L value indicates a darker tone). The value was similar in the two cultivars at an early stage (stage 1), but became substantially higher in AD and lower in NW, respectively, at advanced stages (a high value indicates greater intensity in redness). These results indicated that the accumulation of pigments differed between the two cultivars at all capitulum developmental stages, and that pigments were present in NW at low concentrations at early developmental stages.

### 3.2. Identification and Quantification of Anthocyanins and Other Flavonoids

Given that derivatives of cyanidins are the predominant pigments responsible for pink, purple, and red coloration in the florets of chrysanthemums [20,30], we investigated the contents of anthocyanins and other flavonoids by HPLC-DAD-ESI-MS analysis using samples from plants cultivated in 2014.

**Figure 2:**
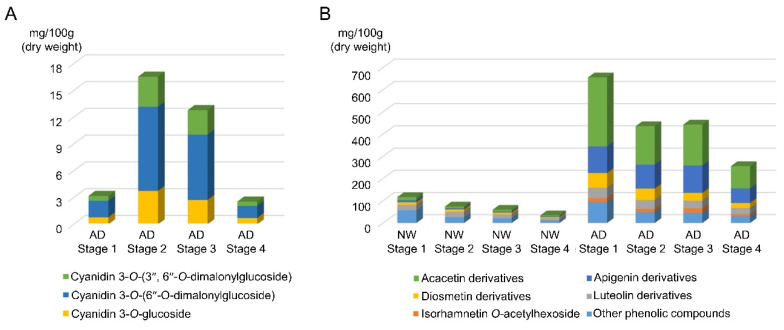
Types and amounts of phenolic compounds at four capitulum developmental stages in “Noble Wine” (NW) and “ARTI-Dark Chocolate” (AD). (**A**) Identities and amounts of anthocyanins. (**B**) Types and amounts of other flavonoids and phenolic compounds. The amounts of compounds were determined based on HPLC peak areas relative to those of standard compounds which were in known amounts and co-analyzed (cyanidin and quercetin for flavonoids and anthocyanin, respectively). Stages 1 to 4 represent different ray floret developmental stages. Flavonoids were subdivided according to aglycone type.

HPLC-DAD-ESI-MS analysis performed at 515 nm to detect anthocyanins in the ray floret corolla of AD identified three cyanidin derivatives, namely cyanidin 3-*O*-glucoside, cyanidin 3-*O*-(6″-*O*-malonylglucoside), and cyanidin 3-*O*-(3″,6″-*O*-dimalonylglucoside), together with three additional phenolic compounds with maximum absorbances observed between 326 and 347 nm (Table 1 and Table 2, Supplementary Figure S1). The concentration of cyanidin 3-*O*-(6″-*O*-malonylglucoside) was highest (53.3–58.3% depending on the developmental stage), followed by that of cyanidin 3-*O*-glucoside (21.4–27.8%) and cyanidin 3-*O*-(3″,6″-*O*-dimalonylglucoside) (18.8–21.4%). The amount of all three compounds increased rapidly with the exsertion of the ray florets (stage 2), showing the highest values at stages 2 and 3, and then highly decreased with the opening of disc florets at stage 4 (Figure 2A). The amounts of all three anthocyanins in the ray floret corolla of NW were below the detectable concentration (Table 2).

**Figure 3:**
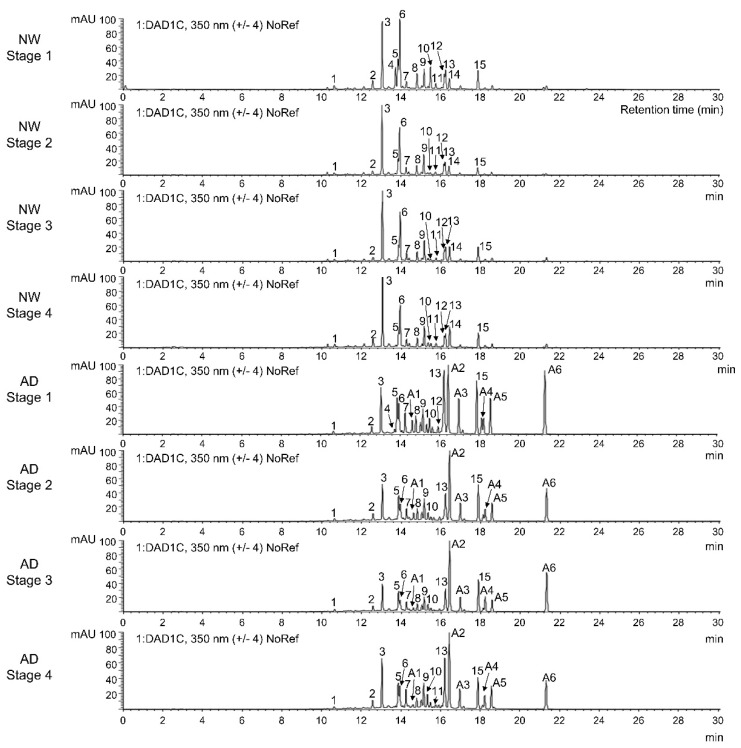
HPLC chromatogram (wavelength 350 nm) obtained from analysis of the ray floret corolla at four capitulum developmental stages in “Noble Wine” (NW) and “ARTI-Dark Chocolate” (AD). The number above each peak corresponds to those listed in Table 1.

The contents of other flavonoids were investigated by HPLC-DAD-ESI-MS analysis under ultraviolet light at 350 nm. We detected a total of 21 phenolic compounds in NW or AD, which included 15 flavonoids that were subclassified as 14 flavones (derivatives of luteolin, apigenin, diosmetin, and acacetin) and one flavonol (an isorhamnetin derivative). Five flavone derivatives, including three acacetin derivatives and one flavonol derivative (peak numbers A1–A6), were specific to AD, whereas a hydrocinnamic acid (caffeoyl-dihydroxyphenyllactoyl-tartaric acid; peak number 14) was detected only in NW (Table 1 and Table 2, Figure 3). The total amount of flavonoids differed among the developmental stages in both AD and NW (Table 2, Figure 2B). It was highest at stage 1 and decreased at subsequent stages. At all stages, the ray floret corolla of AD contained 8.0 (stage 2) to 10.3 times (stage 3) higher flavonoid contents than those of NW (*p*-value < 0.01 in all stages). However, this difference was greatly reduced when the amount of non-flavonoid phenolic compounds were compared; it was only 1.6 (stage 1) to 2.5 times (stage 4) higher in AD than in NW (Table 2, Figure 2B). Thus, the proportion of flavonoid compounds among the total phenolic compounds was much higher in AD (86.6–89.7%) than in NW (51.1–64.8%) (*p*-value < 0.01 in all stages; Table 2; Figure 2B). When comparing the proportions of compounds according to the type of flavonoids, the proportion of acacetin derivatives was highest in AD, followed by that of derivatives of apigenin (44.5–55.3%), diosmetin (8.8–13.4%), luteolin (8.6–12.7%), and isohamnetin (3.2–5.7%). Unlike AD, NW showed the highest proportion of luteolin derivatives (41.1–50.7%), followed by derivatives of diosmetin (18.9–21.1%), acacetin (14.3–23.3%), and apigenin (12.9–16.7%). The amount of each type of flavonoid was 1.6 (luteolin derivatives; stage 2) to 25.1 (acacetin derivatives; stage 2) times higher in AD than in NW, or was detected only in AD (isorhamnetin *O*-acetylhexoside) (*p*-value < 0.01 in all stages Table 2; Figure 2B). Therefore, we concluded that a broad range of flavonoids were simultaneously and highly accumulated in AD in comparison with the contents of NW.

### 3.3. Transcriptome Sequencing and Analysis of Flavonoid Biosynthetic Gene Expression

We assumed that the gene expression responsible for metabolic differences could be detected at stage 1 of ray floret development or earlier because NW and AD showed distinct differences in ray floret color and flavonoid content from stage 1 (Figure 1, Table 2). Therefore, we performed a transcriptome analysis of the ray floret corolla of NW and AD at stage 1 that were obtained from plants which were cultivated together with those used for metabolic analysis in 2014. A total of 5.4 and 4.3 Gb clean reads were obtained for NW and AD, respectively. The sequences were assembled into 103,470 unigenes with an average length of 567 bp and N_50_ of 757 bp. Among these unigenes, 56,966 and 58,467 were expressed at fragment per kilobase of transcripts per million mapped reads (FPKM) values higher than 1.0 in NW and AD, respectively (Supplementary Table S1). The expression levels of unigenes determined by transcriptome analysis were validated by qRT-PCR analysis for 48 unigenes showing various expression levels (Supplementary Figure S2A, Supplementary Table S2). The Pearson correlation coefficient (*r*) for correlation between gene expression levels observed in the two types of analyses was 0.816 (*p*-value = 1.53 × 10^−12^). We performed an additional qRT-PCR analysis for the same panel of unigenes using samples obtained from plants cultivated in 2019 to estimate the impact of subtle environmental change on gene expression pattern. The *r* value was 0.782 (*p*-value = 5.24 × 10^−11^) for correlation between expression levels determined by transcriptome analysis using samples obtained in 2014 and qRT-PCR analysis using those obtained in 2019 (Supplementary Figure S2B). This was slightly smaller than that obtained in the former analysis. The correlation was significantly high (*r* = 0.898; *p*-value = 5.65 × 10^−18^) between qRT-PCR analyses using samples obtained in 2014 and 2019 (Supplementary Figure S2C). This indicates that subtle changes in the environment slightly affected gene expression, but the effect was not high enough to change the overall pattern of it.

**Figure 4:**
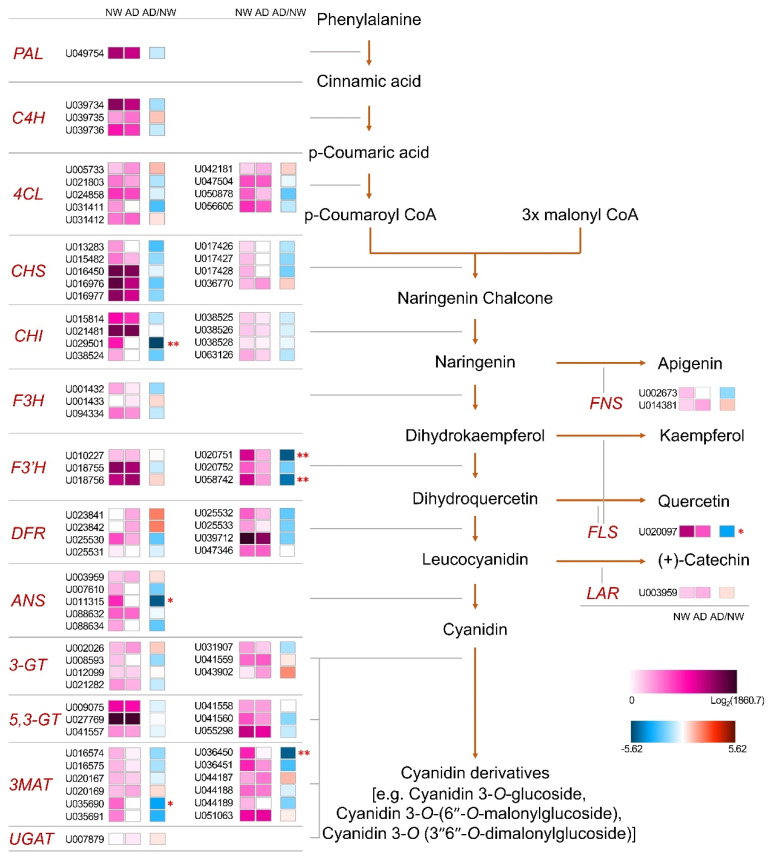
Heat map representing fragments per kilobase of transcript per million mapped reads (FPKM) values for transcriptome sequencing reads of unigenes in the flavonoid biosynthetic pathway in the ray floret corolla (capitulum developmental stage 1) of “Noble Wine” (NW) and “ARTI-Dark Chocolate” (AD). Logarithms of the FPKM + 1 values for NW and AD are represented using squares of different colors in the “NW” and “AD” columns, respectively. Logarithms of the ratios between the FPKM + 1 values for NW and AD are indicated using squares of different colors in the “AD/NW” column. The unigenes showing significant (*p* < 0.05) and highly significant (*p* < 0.001) differences in transcript levels are indicated by “*” and “**”, respectively. *PAL*, phenylalanine ammonia-lyase; *C4H*, cinnamate-4-hydroxylase; *4CL*, 4-coumarate-CoA ligase; *CHS*, chalcone synthase; *CHI*, chalcone isomerase; *F3H*, flavanone 3-hydroxylase; *F3’H*, flavanone 3’-hydroxylase; *DFR*, dihydroflavonol 4-reductase; *ANS*, anthocyanin synthase; *3-GT*, UDP-glucose:anthocyanidin 3-*O*-glucosyltransferase; *3*,*5-GT*, UDP-glucose:anthocyanin 5,3-*O*-glucosyltransferase; *3MAT*, malonyl-coenzyme A:anthocyanin 3-*O*-glucoside-6”-*O*-malonyltransferase; *UGAT*, cyanidin-3-*O*-glucoside 2”-*O*-glucuronosyltransferase; *FNS*, flavone synthase; *FLS*, flavonol synthase; *LAR*, leucoanthocyanidin reductase.

We assigned unigenes to flavonoid biosynthesis pathway genes based on annotations retrieved by BLAST and InterProScan analysis for the comparison of gene expression levels between NW and AD (Figure 4). One (for cyanidin-3-*O*-glucoside 2”-*O*-glucuronosyltransferase (*UGAT*), flavonol synthase (*FLS*), and leucoanthocyanidin reductase (*LAR*)) to 12 (for malonyl-coenzyme A:anthocyanin 3-*O*-glucoside-6”-*O*-malonyltransferase (*3MAT*)) unigenes were assigned to 16 gene categories that covered both early and late flavonoid biosynthetic genes involved in the biosynthesis of anthocyanins, flavonols, flavones, and flavanols. Interestingly, the expression level of these genes was mostly lower in AD than in NW. In particular, one or two unigenes assigned to each of *CHI*, *F3′H*, *ANS*, *MAT*, and *FLS* were significantly or highly significantly suppressed in AD (Figure 4). This result showed that there is no clear relation between gene expression level and flavonoid accumulation in AD.

We performed qRT-PCR analysis using plant samples obtained in 2019 for each unigene that showed the highest expression in AD among the unigenes annotated to each flavonoid biosynthetic gene to check the reproducibility of the expression pattern of flavonoid biosynthetic genes at stage 1 and the possibility that the expression levels of these unigenes are related to flavonoid accumulation (Supplementary Figure S3). At stage 1, expression levels of U024858 (*4CL*), U016450 (*CHS*), U018756 (*F3′H*), and U003959 (*LAR*) were significantly higher in AD, while those of U094334 (*F3H*), U039712 (*DFR*), and U020097 (*FLS*) were higher in NW. In the case of genes showing a higher expression level in AD, the difference in expression level from NW was small, less than 1.5 times, at stage 1. However, at later stages, expression levels of unigenes annotated to *CHS*, *CHI*, *FLS*, and *LAR* were much higher in AD compared to NW (Supplementary Figure S3). This result leaves a possibility that the expression of specific unigenes assigned to the flavonoid biosynthetic pathway partially influences flavonoid accumulation in AD.

### 3.4. Functional Analysis of Differentially Expressed Genes (DEGs)

**Figure 5:**
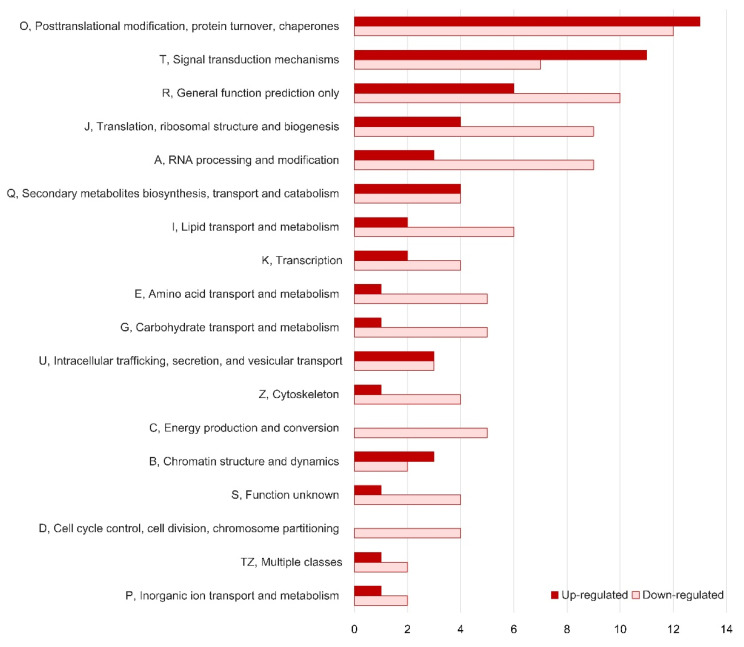
Grouping of differentially expressed genes based on euKaryotic Orthologous Groups (KOG) analysis.

For further analysis of the mechanism of flavonoid accumulation in AD, we screened DEGs and performed a functional classification of them. A total of 295 and 230 DEGs were up- and down-regulated in AD compared with NW, respectively (*p*-value < 0.005; Supplementary Table S3). A gene ontology (GO) analysis classified the DEGs into 41 functional groups (Supplementary Figure S4). The proportion of down-regulated genes was higher than that of up-regulated genes in the majority of functional groups (39 groups) because the proportion of unigenes that could be assigned to one or more GO terms was higher among the down-regulated DEGs (54.8%) than the up-regulated DEGs (29.8%). The number of up-regulated unigenes was higher than the number that were down-regulated among unigenes that were grouped in the “enzyme regulator” category (five and one among the up-regulated and down-regulated unigenes, respectively), whereas the proportion of down-regulated unigenes was relatively higher in functional groups classified as “macromolecular complex” (70.9%), “response to stimuli” (69.6%), and “transporter activity” (68.8%) (Supplementary Figure S4). In a euKaryotic Orthologous Groups (KOG) analysis for further functional classification, 57 and 97 unigenes were classified into 18 functional groups (Figure 5). Among groups containing at least ten unigenes, the category “post-translational modification, protein turnover, chaperones” contained the largest number of both up-regulated and down-regulated unigenes. Up-regulated unigenes were enriched in the category “signal transduction mechanisms”, whereas down-regulated unigenes were predominantly classified in the “translation, ribosomal structure, and biogenesis” and “RNA processing and modification” groups (Figure 5). The assignment of unigenes to Kyoto Encyclopedia of Genes and Genomes (KEGG) pathways was unsuccessful because no pathways contained a notable number of DEGs (more than three unigenes).

**Figure 6:**
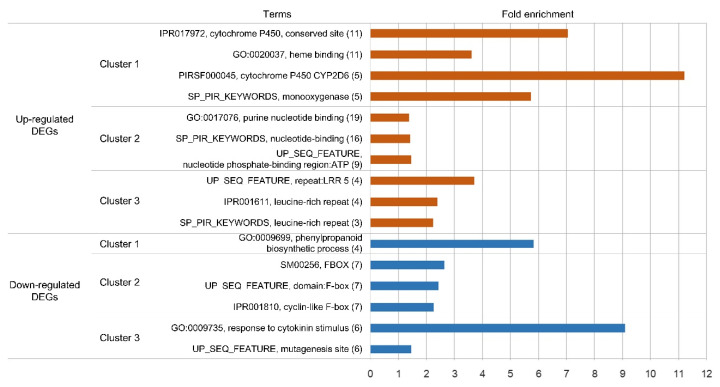
Highest-ranked functional clusters for differentially expressed genes (DEGs) determined using the DAVID gene functional classification tool. In each cluster, only one term showing the highest fold enrichment value was selected from each functional annotation source and shown in this figure. The number of unigenes annotated with each term is shown in parentheses. Information for all terms in these clusters is presented in Tables S4 and S5.

**Figure 7:**
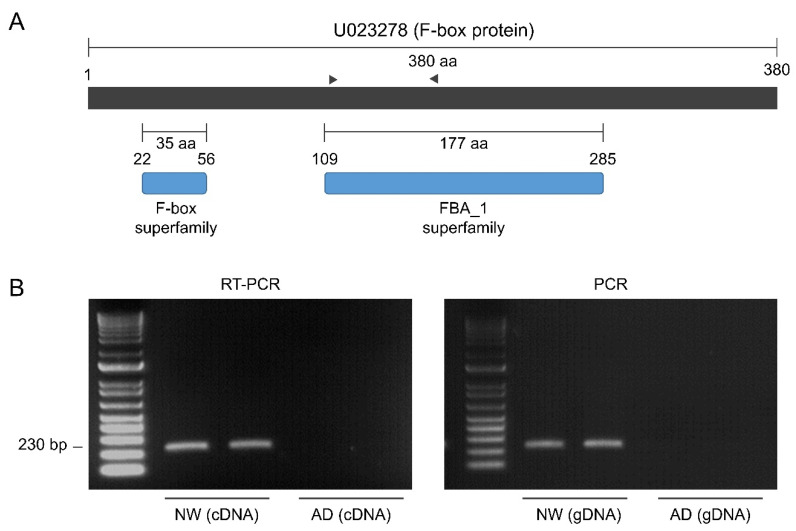
Analysis of a down-regulated unigene U023278 annotated as an F-box protein. (**A**) Predicted functional domains in the F-box protein. The locations of primers used for qRT-PCR and PCR analyses (**B**) are indicated by black triangles. (**B**) Amplification results for qRT-PCR and PCR analyses of U23278.

**Figure 8:**
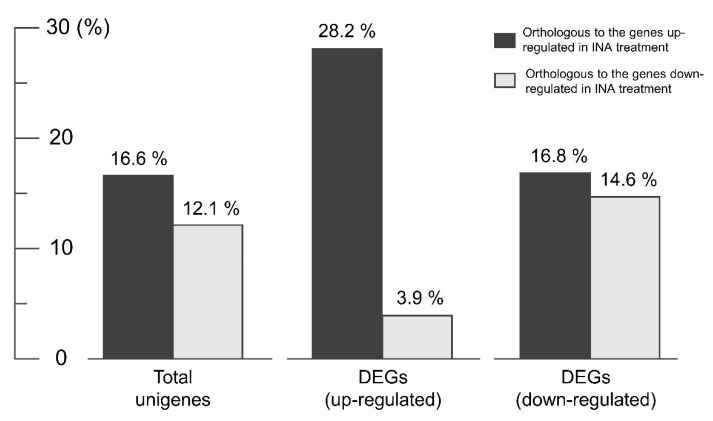
Proportion of total unigenes and differentially expressed genes (DEGs) that showed high similarity with *Arabidopsis* genes reported to be up- or down-regulated in response to salicylic acid (SA) treatment. The list of DEGs was obtained from Jin et al. [48], who applied 2,6-dichloroisonicotinic acid (INA; an analog of SA).

The aforementioned functional analyses provided only superficial information, therefore we conducted clustering and enrichment analysis using the DAVID functional annotation tool, by which classifications based on 14 functional annotation sources were integrated [46]. This analysis was performed for the 226 DEGs (93 up-regulated and 133 down-regulated) that could be assigned with UniProt IDs from a similarity search using the InterProScan tool. We obtained clusters of genes that showed functional similarities for the up-regulated and down-regulated unigenes, respectively (Figure 6, Supplementary Tables S4 and S5). The result for the down-regulated genes was notable because the clusters containing phenylpropanoid pathway genes and F-box protein-coding genes, for which a direct relationship with flavonoid accumulation has been reported by previous studies, were ranked as the first and second most highly enriched clusters, respectively (Figure 6, Supplementary Table S5). The inverse correlation between the expression pattern of phenylpropanoid pathway genes and the flavonoid content shown in Figure 4 was confirmed based on annotation cluster 1. Cluster 2 consisted of seven unigenes that were annotated as F-box proteins. Several F-box proteins are reported to be involved in the degradation of proteins in early steps of the phenylpropanoid biosynthesis pathway, thus knock-out mutation of the responsible genes resulted in the accumulation of a broad range of flavonoids [13,14,15]. Therefore, we performed PCR analysis using primers specific for each F-box gene in cluster 2 to screen for DNA mutation. The amplicon for a unigene (U233278) that coded for the complete sequence of an FBA 1 superfamily F-box protein was not detected in AD by PCR (using genomic DNA as the template) nor by qRT-PCR (using cDNA as the template) (Figure 7). This result indicated that U233278 may contain the DNA mutation that resulted from mutagenesis. The third enriched cluster included genes involved in responses to hormone stimuli, especially cytokinin stimuli (Figure 7, Supplementary Table S5). In the analysis of up-regulated unigenes, the most highly enriched clusters contained genes for cytochrome P450 proteins, purine nucleotide-binding proteins, and leucine-rich repeat (LRR)-containing proteins (Figure 7, Supplementary Table S4). It is well known that cytochrome P450 proteins are involved in the synthesis of secondary metabolites and plant defense mechanisms [47] and a gene encoding an LRR-containing protein in the third enriched cluster was annotated as a defense-related gene. Therefore, we investigated the co-relationship between the up-regulated unigenes and genes responsive to salicylic acid (SA), which is an important signaling molecule involved in defense against pathogens. We compared the list of *Arabidopsis* genes showing the highest similarity (in amino acid sequences) to each DEG in AD with that of genes screened as DEGs in response to treatment with 2,6-dichloroisonicotinic acid (INA; an analog of SA) in *Arabidopsis* by Jin et al. [48]. The up-regulated unigenes of AD included a substantially higher and lower proportion of genes orthologous to *Arabidopsis* genes up- and down-regulated in response to SA treatment, respectively, in comparison to the total and down-regulated unigenes of AD (Figure 8). This result implied the possibility of cross-talk between flavonoid biosynthesis and the defense mechanism in AD.

## 4. Discussion

In chrysanthemum, anthocyanins and flavonoids have received substantial research attention as determinants of flower color and substances of pharmacological efficacy, respectively [23,30,49]. The mutant cultivar AD is a highly suitable resource for the elucidation of the mechanism of the accumulation of high flavonoid content and the determination of targets for the manipulation of flavonoid content in chrysanthemum because the factors specifically associated with flavonoid content can be analyzed directly by comparative analysis with a parental cultivar.

The flavonoid content of AD was not unusual in comparison with those of other chrysanthemum cultivars analyzed in previous studies, although different patterns of glycosylation were detected in several compounds. The three cyanidin derivatives, and most derivatives of apigenin, luteolin, acacetin, and chrysoeriol detected in AD, have been detected in other analyzed chrysanthemum cultivars [20,30,49]. The characteristic feature of AD was a dramatic increase in the content of a broad range of flavonoids, including colorless flavonoids, as well as anthocyanins. Chen et al. [30] showed that the flavonoid content of a pink-rayed chrysanthemum cultivar (H5) was lower than that of a white-rayed cultivar, although anthocyanins were accumulated specifically in H5. An increase in anthocyanin contents associated with a decline in the accumulation of other flavonoids, the synthesis of which is competitive with that of anthocyanin for common precursors, has also been detected in other floricultural crops, such as dahlia [50], grape hyacinth [51], carnation, and petunia [52]. However, dramatic increases in anthocyanin and other flavonoid contents in AD implied that the factor responsible for flavonoid accumulation might be associated with the regulation of the accumulation of the common precursor(s) rather than influence the competition between the synthesis of anthocyanins and other flavonoids. Considering that the increase in flavonoid content was much higher than that of other phenolic compounds in AD (Figure 2B), this factor might be involved in regulating the accumulation of compounds synthesized upstream of the flavonoid biosynthetic pathway (e.g., naringenin chalcone and nargenin), which is separate from that of other phenolic compounds.

The transcriptional regulation of genes in the flavonoid biosynthetic pathway by transcription factors (e.g., R2R3-MYB proteins, the MBW complex) is the most extensively studied mechanism for anthocyanin accumulation [6]. In recent transcriptome analyses focusing on the accumulation of flavonoids [23] and the light-induced biosynthesis of anthocyanins [22] in chrysanthemum, changes in the expression profile of flavonoid biosynthetic genes were detected and, in turn, their regulation by transcription factors was elucidated as the underlying mechanism. Specific up-regulation of *DFR* and *3GT* transcription has been observed in a pink-rayed chrysanthemum cultivar [30]. The present results are unlikely to support this mechanism because the expression levels of most flavonoid biosynthetic genes were similar between NW and AD or lower in AD at stage 1 when there was a clear difference already in the flavonoid content between the two cultivars. This opposite relationship between the accumulation of anthocyanins or flavonoids and the expression of their biosynthetic genes has also been detected in other crops [51,52]. Negative feedback from the excessive accumulation of flavonoids might be a possible mechanism, although it has not been investigated. Unlike most other unigenes assigned to flavonoid biosythetic genes, several unigenes with high expression levels in AD showed higher expression levels in AD than NW at stage 1, although the differences were not large (Supplementary Figure S3). In addition, the expression levels of some unigenes assigned to *CHS*, *CHI*, *FLS*, and *LAR* became much higher in AD compared to NW at later stages, which might be related to a high accumulation of anthocyanins at stages 2 and 3. However, considering that the total flavonoid content in AD was the highest at stage 1, the moderate increase in the expression of specific unigenes at stage 1 might explain the mechanism of flavonoid accumulation only partially. The regulation of the transport or storage of flavonoids is an additional potential mechanism for flavonoid accumulation, although no supportive evidence was obtained from the current DEG analysis.

We suggest post-translational regulation as a possible mechanism for flavonoid accumulation because (i) a quantitative increase was detected simultaneously in a broad range of flavonoids, including colorless flavonoids by metabolic analysis in AD, implying the up-regulation of early biosynthetic gene(s) and (ii) the enrichment of genes encoding proteins involved in post-translational regulation (notably F-box proteins) among down-regulated DEGs, without the clear transcriptional up-regulation of flavonoid biosynthetic genes, was observed by transcriptome analysis. Increasing evidence for the involvement of the post-translational regulation of flavonoid biosynthetic genes, especially early flavonoid biosynthetic genes, in the determination of flavonoid content, has been reported for many crops [13,14,15,53,54]. Gu et al. [54] reported that CHS, which catalyzes an early step in the flavonoid biosynthetic pathway, contained the largest number of ubiquitination sites among genes in the same pathway. In addition, Zhang et al. [14,15] showed that PAL, the enzyme responsible for the first step of the phenylpropanoid pathway, is a common target of four F-box proteins, thus the knock-out mutation of genes encoding these proteins results in the accumulation of a broad range of flavonoids. We also cannot rule out the possibility that post-translational regulation by DEGs might target proteins that are not included in the flavonoid biosynthetic pathway but could be indirectly involved in flavonoid accumulation. For example, a recent study showed that an F-box protein which was highly expressed during anthocyanin accumulation in colored wheat interacted with aquaporin PIP1, which has been reported to be related with anthocyanin accumulation in *Arabidopsis* [55]. When it is considered that radiation-induced mutations usually result in the dysfunction of several genes [56], dysfunction of the post-translational regulation involving F-box proteins may be caused by mutation of the genes that regulate the expression of F-box genes or the direct mutation of F-box gene(s). Both mechanisms are feasible because both the down-regulation of several F-box genes and the mutation of an F-box gene were detected in the present analysis. Further investigation of the degradation of proteins in the flavonoid biosynthetic pathway and the interactions of these proteins with candidate F-box proteins (products of down-regulated or mutated F-box genes) is required for the aforementioned hypotheses. The up-regulation of genes encoding cytochrome P450 proteins is also a possible mechanism because a primary role of these proteins is the synthesis of secondary metabolites [47]. Indeed, a cytochrome P450 protein encoded by an up-regulated unigene (U059946) was annotated as geraniol 8-hydroxylase, which catalyzes the hydroxylation of naringenin to form a flavanone, eriodictyol [57]. Thus, in AD, the mutation might have been induced in upstream regulatory elements that affect the cross-talk with defense responses, considering that an additional role of P450 proteins is in defense responses and the up-regulation of the putative defense-responsive genes observed in AD. It will be also interesting to investigate the response to pathogens in AD to determine the relationship between flavonoid biosynthesis and disease resistance. Previous research showed that a red mutant line of cotton, which accumulates flavonoids, had an increased resistance to a fungal pathogen [58].

In conclusion, the simultaneous accumulation of diverse flavonoids and the alteration in gene expression patterns were revealed by metabolic and transcriptomic analyses, respectively, in a novel chrysanthemum mutant cultivar. Considering the results from both approaches, we suggest post-translational regulation as one potential mechanism for the observed increase in flavonoid content in the mutant cultivar. The characterized plant material and information on the transcriptome profile from the present study will contribute to future research on the regulation and manipulation of flavonoid biosynthesis in chrysanthemum.

## Figures and Tables

**Table 1:** Peak assignments of the aqueous methanol extract from the ray floret corolla of chrysanthemums “Noble Wine” and “ARTI-Dark Chocolate”.

Peak Number	tR (min)	M + H^+^/M + H^−^ (*m*/*z*)	UV Λmax (nm)	Identification
350 nm				
1	10.74	-/353	326	3-Caffeyolquinic acid [42] ^x^
2	12.67	595/563	338	6,8-C,C-diglucosylapigenin [42] ^x^
3	13.06	449/447	348	Luteolin-7-*O*-glucoside [42] ^x^
4	13.84	-/515	326	3,4-Di-caffeoylquinic acid [42] ^x^
5	13.88	-/515	326	1,4-Di-caffeoylquinic acid [42] ^x^
6	13.96	-/515	326	3,5-Di-caffeoylquinic acid [42] ^x^
7	14.04	433/431	332	Apigenin-7-*O*-glucoside [42] ^x^
A1 ^z^	14.36	463/461	nd ^y^	Trihydroxymethoxyflavone glucoside [42] ^x^
8	14.75	477/475	348	Diosmetin-7-*O*-glucuronide [42] ^x^
9	15.17	549/547	348	Diosmetin-7-*O*-6″-malonylglucoside [42] ^x^
10	15.50	-/529	nd	4-Caffeoyl-5-feruloylquinic acid isomer [42] ^x^
11	15.76	287/285	348	Luteolin [42] ^x^
12	16.16	271/269	334	Apigenin [42] ^x^
13	16.17	533/531	334	Acacetin-7-*O*-6″-malonylgactoside [42] ^x^
A2	16.25	563/-	338	Apigenin-8-*C*-hexoside-7-*O*-pentoside [43] ^w^
14	16.26	-/491	nd	Caffeoyl-dihydroxyphenyllactoyl-tartaric acid [43] ^w^
A3	17.06	533/-	325	Acacetin-malonylglucoside [44] ^x^
15	17.88	619/-	326	Acacetin-7-*O*-(3,6-*O*-dimalonyl)-β-D-glucopyranoside [30] ^w^
A4	18.24	-/519	358	Isorhamnetin *O*-acetylhexoside [44] ^w^
A5	18.66	547/-	326	Acacetin-7-*O*-(3-*O*-malonyl)-β-D-glucuronopyranoside [30] ^w^
A6	21.35	285/283	330	Acacetin [42] ^x^
515 nm				
A7	10.33	449/625	516	Cyanidin 3-*O*-glucoside [45] ^x^
A8	11.338	535/529	518	Cyanidin 3-*O*-(6″-*O*-malonylglucoside) [45] ^x^
A9	11.99	621/571	518	Cyanidin 3-*O*-(3″6,″-*O*-dimalonylglucoside) [30] ^x^
A10	12.251	549/571	347	Chrysoeriol 7-*O*-malonylglucoside [30] ^x^
A11	12.83	635/677	342	Acetylated luteolin hexoxyl-rhamnoside [29] ^w^
6	13.96	-/515	326	3,5-Di-caffeoylquinic acid [42] ^x^

^w^ Identification was confirmed by comparison with standards or positively identified compounds in reference plant samples. References are presented. ^x^ Previously reported in chrysanthemum florets. References are presented. ^y^ nd: Not determined. ^z^ Peaks specifically detected in “ARTI-Dark Chocolate” are numbered A1–A6.

**Table 2:** Contents of flavonoid compounds in the ray floret corolla of chrysanthemums “Noble Wine” (NW) and “ARTI-Dark Chocolate” (AD) determined by liquid chromatography/mass spectrometry (mg/100 g (dry weight)) ^z^.

	NW Stage 1	NW Stage 2	NW Stage 3	NW Stage 4	AD Stage 1	AD Stage 2	AD Stage 3	AD Stage 4
350 nm								
1 ^y^	0.5 ± 0.001 ^ax^	0.3 ± 0.001 ^b^	0.3 ± 0.004 ^c^	0.3 ± 0.004 ^c^	1.0 ± 0.004 ^d^	0.6 ± 0.001 ^e^	0.6 ± 0.001 ^f^	0.4 ± 0.001 ^g^
2	2.3 ± 0.007 ^a^	0.8 ± 0.002 ^b^	0.7 ± 0.002 ^c^	1.2 ± 0.008 ^d^	8.0 ± 0.005 ^e^	8.4 ± 0.001 ^f^	7.9 ± 0.001 ^g^	5.3 ± 0.003 ^h^
3	22.4 ± 0.046 ^a^	23.6 ± 0.001 ^b^	17.5 ± 0.003 ^c^	9.5 ± 0.011 ^d^	47.0 ± 0.011 ^e^	39.8 ± 0.001 ^f^	34.3 ± 0.014 ^g^	26.7 ± 0.018 ^h^
4	12.7 ± 0.008 ^a^	-	-	-	3.1 ± 0.002 ^b^	-	-	-
5	9.3 ± 0.012 ^a^	5.2 ± 0.003 ^b^	3.6 ± 0.001 ^c^	2.3 ± 0.007 ^d^	39.3 ± 0.007 ^e^	26.4 ± 0.004 ^f^	25.5 ± 0.010 ^g^	14.0 ± 0.004 ^h^
6	23.3 ± 0.008 ^a^	16.5 ± 0.001 ^b^	12.6 ± 0.001 ^c^	5.8 ± 0.008 ^d^	33.1 ± 0.019 ^e^	18.4 ± 0.005 ^f^	14.4 ± 0.007 ^g^	12.2 ± 0.009 ^h^
7	2.1 ± 0.002 ^a^	2.4 ± 0.001 ^b^	1.8 ± 0.001 ^c^	1.0 ± 0.006 ^d^	24.8 ± 0.005 ^e^	15.0 ± 0.004 ^f^	12.4 ± 0.006 ^g^	11.7 ± 0.008 ^h^
8	5.3 ± 0.007 ^a^	3.1 ± 0.001 ^b^	2.4 ± 0.002 ^c^	1.3 ± 0.003 ^d^	20.8 ± 0.012 ^e^	15.0 ± 0.004 ^f^	10.4 ± 0.005 ^g^	6.8 ± 0.005 ^h^
A1	-	-	-	-	16.1 ± 0.009 ^a^	10.1 ± 0.003 ^b^	3.9 ± 0.002 ^c^	2.4 ± 0.002 ^d^
9	6.6 ± 0.008 ^a^	7.1 ± 0.002 ^b^	5.2 ± 0.004 ^c^	2.9 ± 0.003 ^d^	29.2 ± 0.004 ^e^	26.7 ± 0.007 ^f^	20.6 ± 0.005 ^g^	13.2 ± 0.003 ^h^
10	7.6 ± 0.004 ^a^	0.7 ± 0.001 ^b^	0.3 ± 0.002 ^c^	0.4 ± 0.001 ^d^	17.6 ± 0.007 ^e^	4.1 ± 0.001 ^f^	5.2 ± 0.001 ^g^	3.9 ± 0.003 ^h^
11	2.1 ± 0.001 ^a^	0.9 ± 0.001 ^b^	0.6 ± 0.003 ^c^	0.5 ± 0.002 ^d^	-	-	-	1.9 ± 0.001 ^e^
12	4.9 ± 0.005 ^a^	3.5 ± 0.001 ^b^	2.5 ± 0.002 ^c^	1.5 ± 0.001 ^d^	8.1 ± 0.005 ^e^	-	-	-
13	6.5 ± 0.00 1 ^a^	4.5 ± 0.002 ^b^	3.7 ± 0.001 ^c^	1.9 ± 0.003 ^d^	90.5 ± 0.042 ^e^	41.9 ± 0.011 ^f^	37.2 ± 0.019 ^g^	33.8 ± 0.009 ^h^
A2	-	-	-	-	78.9 ± 0.047 ^a^	84.1 ± 0.016 ^b^	103.1 ± 0.021 ^c^	48.6 ± 0.031 ^d^
14	3.7 ± 0.005 ^a^	3.5 ± 0.001 ^b^	4.3 ± 0.001 ^c^	3.4 ± 0.003 ^d^	-	-	-	-
A3	-	-	-	-	40.8 ± 0.020 ^a^	21.2 ± 0.002 ^b^	20.5 ± 0.010 ^c^	12.0 ± 0.008 ^d^
15	7.4 ± 0.001 ^a^	2.4 ± 0.002 ^b^	4.3 ± 0.002 ^c^	2.4 ± 0.001 ^d^	59.9 ± 0.013 ^e^	44.2 ± 0.008 ^f^	46.4 ± 0.023 ^g^	19.9 ± 0.008 ^h^
A4	-	-	-	-	17.8 ± 0.010 ^a^	14.2 ± 0.001 ^b^	20.7 ± 0.010 ^c^	8.7 ± 0.006 ^d^
A5	-	-	-	-	38.8 ± 0.022 ^a^	21.4 ± 0.003 ^b^	16.5 ± 0.008 ^c^	14.1 ± 0.010 ^d^
A6	-	-	-	-	80.7 ± 0.025 ^a^	44.5 ± 0.012 ^b^	63.7 ± 0.026 ^c^	20.5 ± 0.014 ^d^
515 nm								
A7	-	-	-	-	0.79 ± 0.005 ^a^	3.75 ± 0.007 ^b^	2.74 ± 0.005 ^c^	0.71 ± 0.011 ^d^
A8	-	-	-	-	1.86 ± 0.036 ^a^	9.43 ± 0.152 ^b^	7.32± 0.020 ^c^	1.36 ± 0.008 ^d^
A9	-	-	-	-	0.54 ± 0.013 ^a^	3.37 ± 0.022 ^b^	2.74 ± 0.018 ^c^	0.48 ± 0.004 ^a^
A10	-	-	-	-	0.75 ± 0.011 ^a^	3.63 ± 0.004 ^b^	2.40 ± 0.018 ^c^	0.63 ± 0.016 ^d^
A11	-	-	-	-	0.42 ± 0.001 ^a^	2.62 ± 0.007 ^b^	1.84 ± 0.009 ^c^	0.39 ± 0.013 ^a^
6	-	-	-	-	0.11 ± 0.001 ^a^	0.38 ± 0.014 ^b^	0.30 ± 0.002 ^c^	0.11 ± 0.005 ^a^

^x^ Values are the mean ± standard error (*n* = 3). Different letters indicate statistical differences analyzed by one-way ANOVA and Duncan’s multiple range test. ^y^ Peak number listed in Table 1. ^z^ Quercetin and cyanidin were used as a standard for quantification in analysis at 350 nm and 515 nm, respectively.

## Supplementary Materials

The following are available online at https://www.mdpi.com/2073-4425/11/8/865/s1, Figure S1: HPLC chromatogram (wavelength 515 nm) obtained from analysis of the ray floret corolla at four capitulum developmental stages in AD, Figure S2: Correlation between expression levels of unigenes determined by a transcriptomic analysis and two qRT-PCR analyses using samples obtained in two different years, Figure S3: Expression of unigenes annotated as flavonoid biosynthetic genes at four flower stages in Noble Wine (NW) and ARTI-Dark Chocolate (AD), Figure S4: Classification of differentially expressed genes (DEGs) by gene ontology (GO) analysis based on assignment of level 2 GO terms to DEGs, Table S1: Transcriptome sequencing and unigene assembly information, Table S2: Comparison of expression levels of differentially expressed genes (DEGs) determined by transcriptome sequencing and qRT-PCR analysis, respectively, Table S3: Expression levels of differentially expressed genes, Table S4: Three highest-ranked functional clusters of up-regulated genes determined by DAVID gene functional classification tool, Table S5: Three highest-ranked functional clusters of down-regulated genes determined using the DAVID gene functional classification tool.

## Abbreviations

HPLC-DAD-ESI-MS	high-performance liquid chromatography coupled with photodiode array detection and electrospray ionization mass spectrometry
CHS	chalcone synthase
CHI	chalcone isomerase
bHLH	basic helix-loop-helix
KFB	Kelch motif-containing F-box
PAL	phenylalanine ammonia-lyase
FPKM	fragment per kilobase of transcripts per million mapped reads
C4H	cinnamate-4-hydroxylase
4CL	4-coumarate-CoA ligase
F3H	flavanone 3-hydroxylase
F3’H	flavanone 3’-hydroxylase
DFR	dihydroflavonol 4-reductase
ANS	anthocyanin synthase
3-GT	UDP-glucose:anthocyanidin 3-*O*-glucosyltransferase
3,5-GT	UDP-glucose:anthocyanin 5,3-*O*-glucosyltransferase
3MAT	malonyl-coenzyme A:anthocyanin 3-*O*-glucoside-6”-*O*-malonyltransferase
UGAT	cyanidin-3-*O*-glucoside 2”-*O*-glucuronosyltransferase
FNS	flavone synthase
FLS	flavonol synthase
LAR	leucoanthocyanidin reductase
DEG	differentially expressed gene
GO	gene ontology
KEGG	Kyoto encyclopedia of genes and genomes
KOG	eukaryotic orthologous groups
ELSD	evaporative light scattering detector
CDS	protein coding sequence
