# Supplementary material for: Dramatic Increase in Content of Diverse Flavonoids Accompanied with Down-Regulation of F-Box Genes in a Chrysanthemum (Chrysanthemum × morifolium (Ramat.) Hemsl.) Mutant Cultivar Producing Dark-Purple Ray Florets
Source: Genes (Basel). 2020 Jul 30;11(8):865. doi: 10.3390/genes11080865 (PMC7464468; doi:10.3390/genes11080865)
Supplement: Supplementary file 1 [file genes-11-00865-s001.pdf]

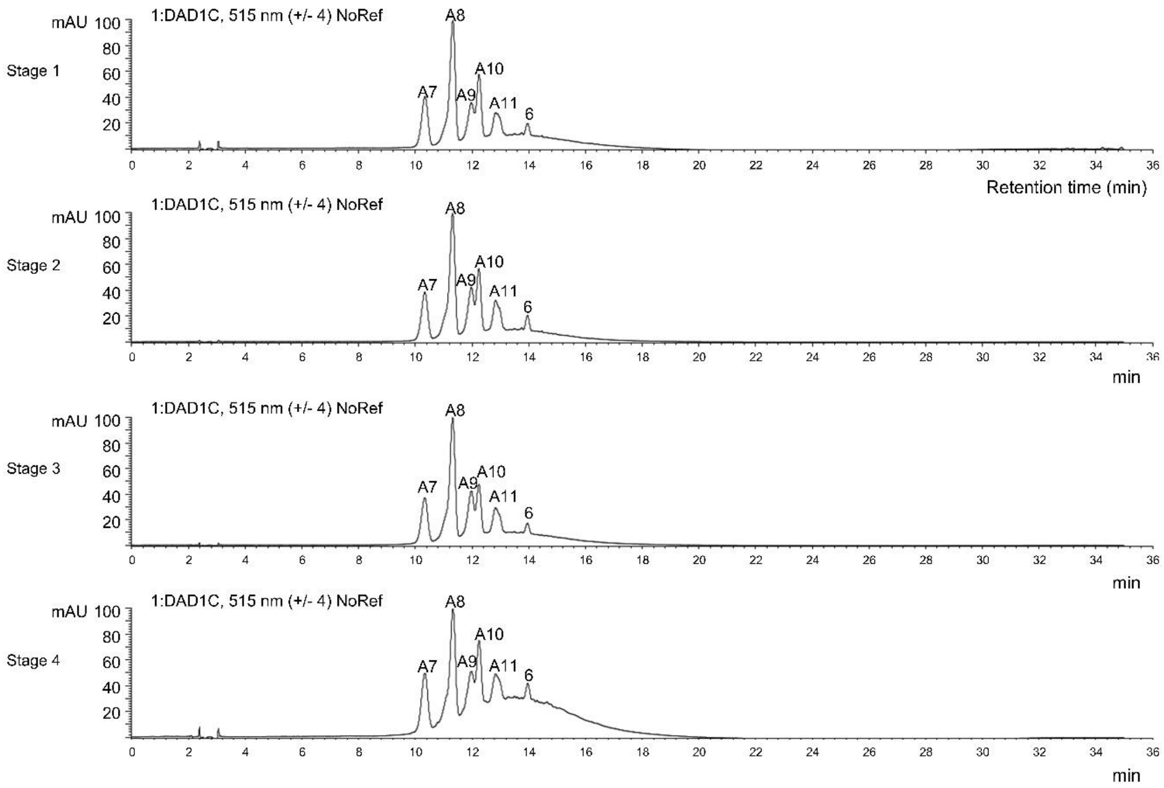

Supplementary Figure S1. HPLC chromatogram (wavelength 515 nm) obtained from analysis of the ray floret corolla at four capitulum developmental stages in AD. Number above each peak correspond to that in table 1.

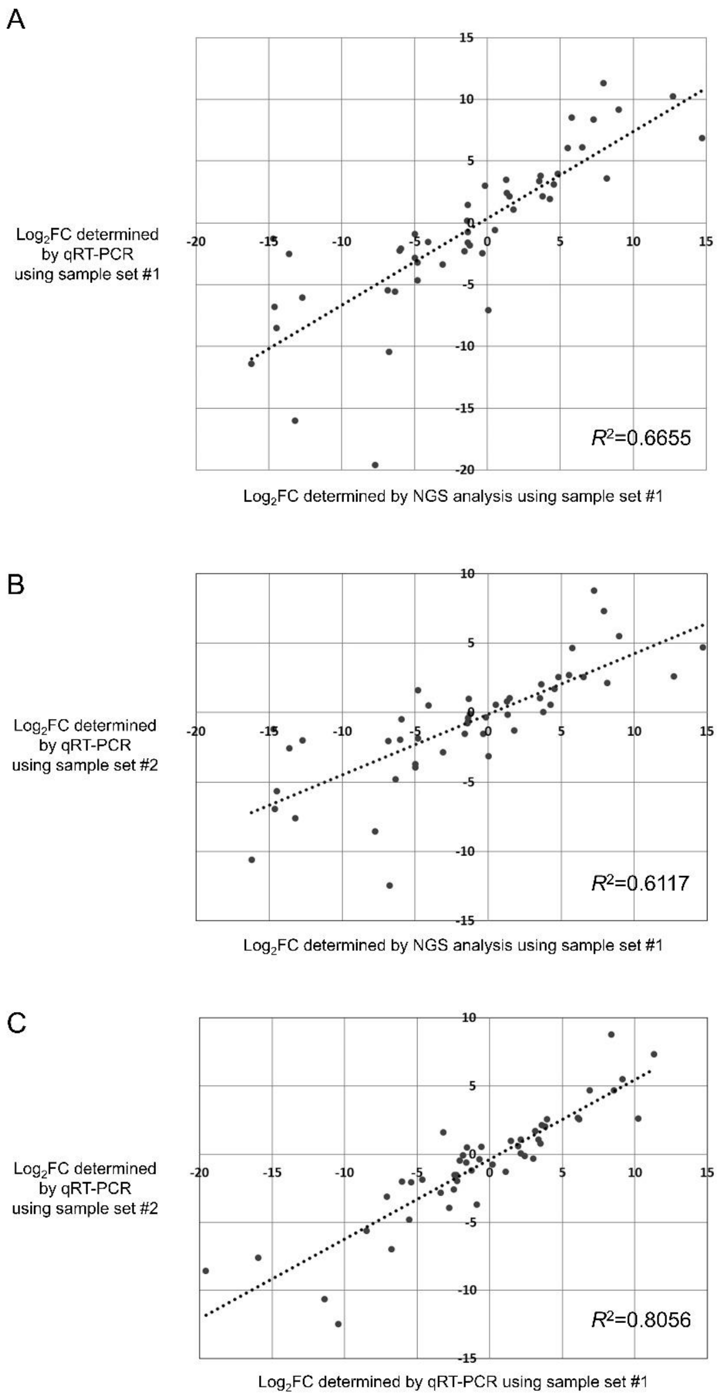

Supplementary Figure S2. Correlation between expression levels of unigenes determined by a transcriptomic analysis and two qRT-PCR analyses using samples obtained in two different years. This analysis was performed for 48 unigenes listed in supplementary table 2. Log_2FC refers to logarithm (base 2) of the expression level of unigenes in ARTI-Dark Chocolate relative to that in Noble Wine. (**A**) Correlation between results of qRT-PCR and transcriptome analysis using ray florets at stage 1 of obtained in 2014 (**B**) Correlation between results of qRT-PCR using ray florets at stage 1 obtained in 2019 and transcriptome analysis using ray florets at stage 1 obtained in 2014 (**C**) Correlation between results of qRT-PCR using ray florets at stage 1 obtained in 2019 and those at stage 1 obtained in 2014, respectively.

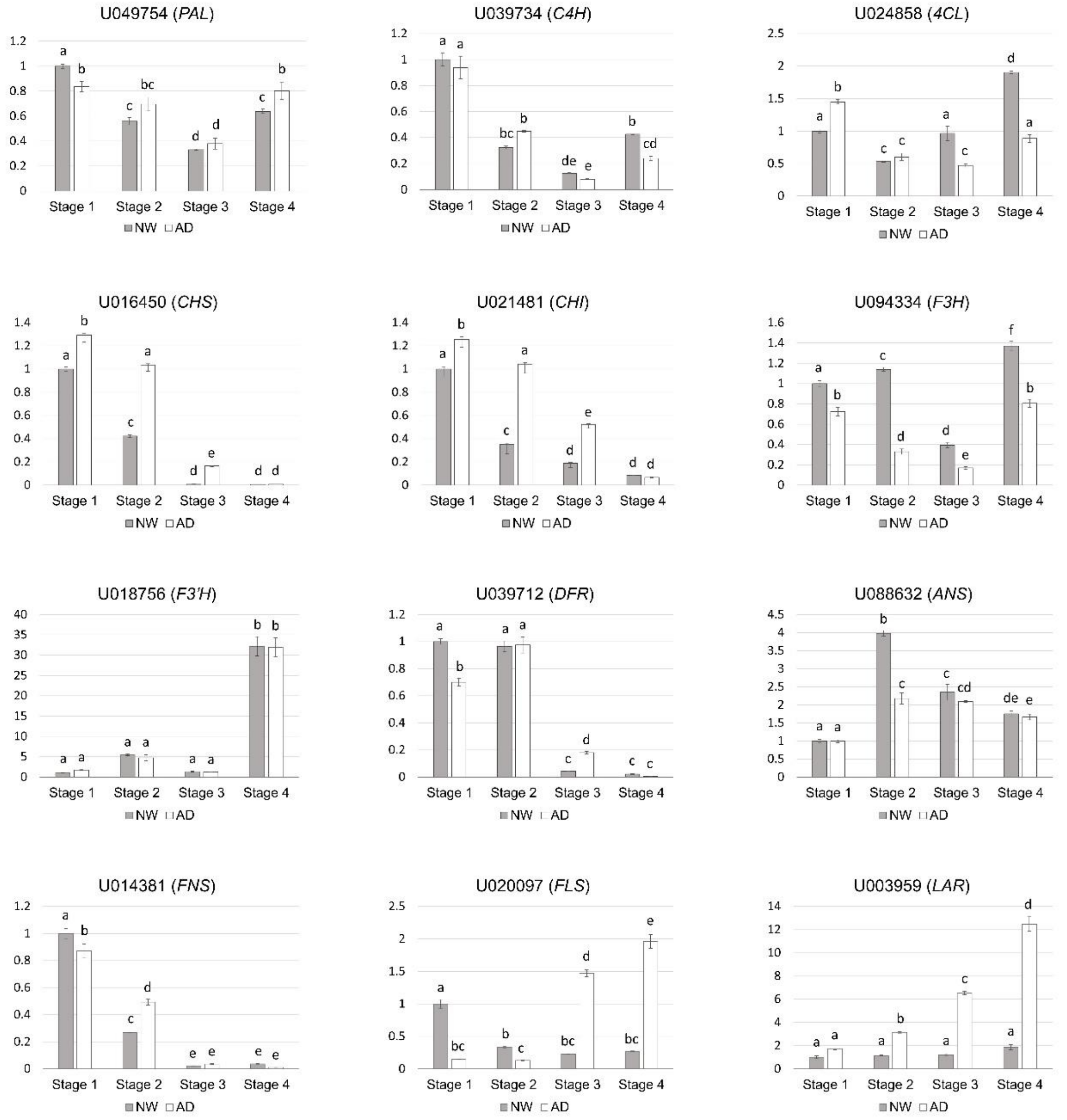

Supplementary Figure S3. Expression of unigenes annotated as flavonoid biosynthetic genes at four flower stages in Noble Wine (NW) and ARTI-Dark Chocolate (AD). Relative expression levels were determined by qRT-PCR analysis. When multiple unigenes were annotated to a single flavonoid biosynthetic gene, the unigene that showed the highest expression level in AD in transcriptome analysis (Figure 4) was selected for this analysis. Error bars indicate ±standard error (n = 3). Different letters above bars in graphs indicate statistical difference analyzed by one-way ANOVA and Duncan's multiple range test.

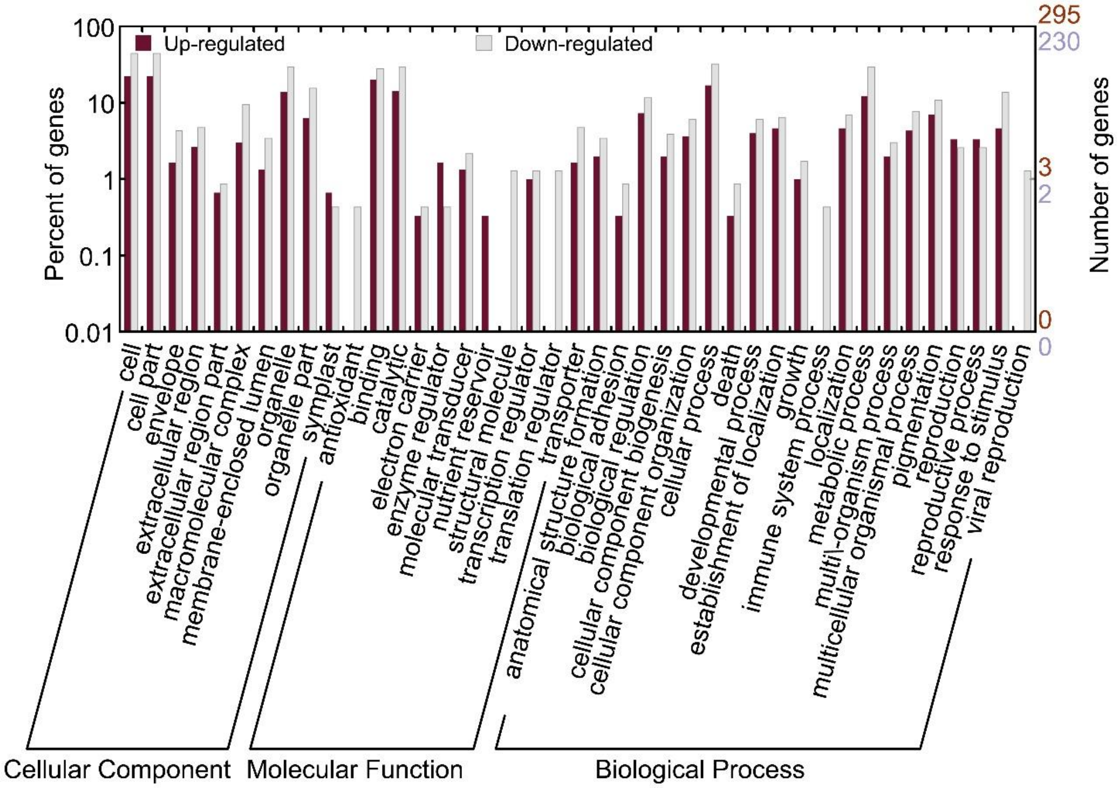

Supplementary Figure S4. Classification of differentially expressed genes (DEGs) by gene ontology (GO) analysis based on assignment of level 2 GO terms to DEGs

Supplementary Table S1. Transcriptome sequencing and unigene assembly information

Total number of clean reads (Total number of base pairs in clean reads)		Number of unigenes	Average length of unigenes	N_{50} of unigenes	Total number of expressed unigenes[Z] (percentage to total number of unigenes)	
NW	AD				NW	AD
53,964,812 (5,377,305,797)	43,514,092 (4,335,046,053)	103,470	567	757	56,966 (55.1%)	58,467 (56.5%)

[Z] Only UniGenes having FPKM value higher than 1.0 were counted

Supplementary Table S2. Comparison of expression levels of differentially expressed genes (DEGs) determined by transcriptome sequencing and qRT-PCR analysis, respectively.

Unigene ID	Description	Transcriptome analysis by NGS				qRT-PCR			
		RPKM in NW	RPKM in AD	Log_2FC	P-value	Sampled in 2014		Sampled in 2019	
						Log_2FC	P-value	Log_2FC	P-value
U004151	Eukaryotic peptide chain release factor subunit 1-3 [Source:SWISS;ACC:P35614]	155.48	0	-16.2	5.91E-06	-11.4	1.70E-04	-10.61	5.01E-03
U013224	Probable receptor-like protein kinase At2g39360 [Source:SWISS;ACC:O80623]	51.02	0	-14.7	1.20E-03	-1.25	6.42E-03	-1.21	5.01E-03
U029501	Chalcone--flavonone isomerase [Source:SWISS;ACC:Q45QI7]	48.24	0	-14.6	2.82E-04	-6.79	1.91E-02	-6.97	3.09E-03
U032284	Glutathione S-transferase DHAR2 [Source:SWISS;ACC:Q9FRL8]	23.72	0	-14.5	9.72E-04	-8.5	8.36E-04	-5.64	1.42E-04
U014436	Cyclin-A2-1 [Source:SWISS;ACC:Q39071]	11.52	0	-13.6	1.29E-03	-2.48	4.89E-03	-2.57	2.27E-03
U016843	-	20.62	0	-13.2	5.87E-03	-15.98	1.73E-02	-7.61	4.41E-04
U011315	Leucoanthocyanidin dioxygenase [Source:SWISS;ACC:Q96323]	35.31	0	-12.7	7.34E-02	-6.03	3.63E-04	-2.02	3.60E-05
U011807	Histone deacetylase 14 [Source:SWISS;ACC:Q941D6]	29.35	0.09	-7.73	1.33E-03	-19.57	3.68E-04	-8.58	7.56E-03
U036450	Malonyl-coenzyme A:anthocyanin 3-O-glucoside-6"-O-malonyltransferase	41.36	0.23	-6.86	1.05E-03	-5.43	7.77E-04	-2.05	7.04E-05

Unigene ID	Description	Transcriptome analysis by NGS				qRT-PCR			
		RPKM in NW	RPKM in AD	Log_2FC	P-value	Sampled in 2014		Sampled in 2019	
						Log_2FC	P-value	Log_2FC	P-value
	{ECO:0000303|PubMed:12481098 } [Source:SWISS;ACC:Q8GSN8]								
U061875	Secoisolariciresinol dehydrogenase [Source:SWISS;ACC:Q94KL8]	238.94	1.45	-6.77	1.59E-04	-10.44	5.97E-05	-12.46	1.26E-03
U061943	Acid phosphatase 1 [Source:SWISS;ACC:P27061]	501.33	4.11	-6.35	3.89E-04	-5.55	4.09E-03	-4.82	1.61E-05
U000039	3-ketoacyl-CoA synthase 17 [Source:SWISS;ACC:Q5XEP9]	60.37	0.61	-6.01	1.75E-03	-2.24	1.14E-02	-1.94	2.18E-05
U015711	Basic blue protein [Source:SWISS;ACC:P00303]	140.33	1.59	-5.95	9.29E-03	-2.05	2.45E-02	-0.49	1.41E-02
U020751	Flavonoid 3'-monooxygenase [Source:SWISS;ACC:Q9SBQ9]	132.27	2.64	-5.01	4.53E-03	-2.8	3.71E-03	-3.94	2.31E-04
U020721	ABC transporter G family member 39 [Source:SWISS;ACC:Q7PC84]	23.68	0.48	-4.98	9.79E-03	-0.87	2.61E-02	-3.7	2.06E-07
U043123	Protein TRANSPARENT TESTA 12 [Source:SWISS;ACC:Q9LYT3]	7.37	0.17	-4.8	4.29E-02	-3.18	1.82E-02	1.6	1.11E-04
U020331	UDP-glycosyltransferase 76C1 [Source:SWISS;ACC:Q9FI99]	173.53	4.04	-4.79	3.24E-03	-4.63	5.46E-04	-1.87	1.03E-03
U029358	1-aminocyclopropane-1-carboxylate oxidase homolog 1 [Source:SWISS;ACC:Q84MB3]	207.11	8.96	-4.07	4.47E-02	-1.56	1.15E-02	0.5	1.17E-02
U020097	Flavonol synthase/flavanone 3-hydroxylase [Source:SWISS;ACC:Q9M547]	218.25	16.59	-3.1	1.72E-02	-3.38	4.37E-03	-2.85	2.16E-03

Unigene ID	Description	Transcriptome analysis by NGS				qRT-PCR			
		RPKM in NW	RPKM in AD	Log_2FC	P-value	Sampled in 2014		Sampled in 2019	
						Log_2FC	P-value	Log_2FC	P-value
U016976	Chalcone synthase 3 [Source:SWISS;ACC:P48392]	889.03	200.47	-1.58	5.43E-01	-2.29	2.08E-03	-1.54	1.79E-03
U025458	Cytochrome P450 98A2 [Source:SWISS;ACC:O48922]	121.08	29.66	-1.4	4.48E-01	0.2	5.23E-01	-0.77	1.19E-03
U027769	Anthocyanidin 5,3-O-glucosyltransferase [Source:SWISS;ACC:Q4R1I9]	1552.95	386.97	-1.37	5.44E-01	-0.72	4.20E-02	-0.39	2.75E-02
U039712	Dihydroflavonol-4-reductase [Source:SWISS;ACC:P51102]	1859.72	475.47	-1.36	5.46E-01	-1.6	2.13E-03	-0.61	6.68E-03
U019144	Probable protein Pop3 [Source:SWISS;ACC:Q9LUV2]	158.99	45.28	-1.35	3.58E-01	1.44	9.60E-04	0.97	8.12E-03
U050684	V-type proton ATPase subunit H [Source:SWISS;ACC:Q9LX65]	217.46	60.87	-1.23	4.46E-01	-1.83	9.56E-05	-0.1	1.40E-01
U023884	Ras-related protein RHN1 [Source:SWISS;ACC:P31583]	167.57	86.77	-0.359	9.42E-01	-2.44	1.16E-03	-1.51	1.38E-05
U018755	Flavonoid 3'-monooxygenase [Source:SWISS;ACC:Q9SBQ9]	454.79	263.35	-0.163	5.62E-01	2.99	1.14E-02	-0.35	4.49E-02
U017796	Gibberellin-regulated protein 10 [Source:SWISS;ACC:Q8LFM2]	65.77	47.98	0.0358	9.40E-01	-7.07	2.32E-04	-3.14	2.85E-03
U021481	Chalcone--flavonone isomerase 2 [Source:SWISS;ACC:A1E261]	636.72	601.17	0.506	6.24E-01	-0.59	1.43E-01	0.56	4.18E-02
U003959	Leucoanthocyanidin dioxygenase [Source:SWISS;ACC:Q96323]	1.62	2.73	1.28	5.94E-01	3.49	2.79E-03	0.8	1.89E-02

Unigene ID	Description	Transcriptome analysis by NGS				qRT-PCR			
		RPKM in NW	RPKM in AD	Log_2FC	P-value	Sampled in 2014		Sampled in 2019	
						Log_2FC	P-value	Log_2FC	P-value
U027507	Shikimate O-hydroxycinnamoyltransferase [Source:SWISS;ACC:Q8GSM7]	41.95	69.05	1.34	2.52E-01	2.42	1.07E-02	-0.13	5.95E-01
U041557	Anthocyanidin 5,3-O-glucosyltransferase [Source:SWISS;ACC:Q4R1I9]	6.87	12.51	1.49	2.47E-01	2.14	5.50E-05	1.06	3.76E-03
U043359	Shikimate O-hydroxycinnamoyltransferase [Source:SWISS;ACC:Q8GSM7]	5.48	13.24	1.77	6.46E-01	1.11	4.21E-03	-1.29	7.27E-02
U013121	Lipid transfer protein EARLI 1 [Source:SWISS;ACC:Q39176]	75.94	626.79	3.57	2.20E-02	3.38	4.03E-02	1.06	2.64E-02
U020473	Aquaporin TIP1-1 [Source:SWISS;ACC:P25818]	15.56	127.76	3.62	3.65E-03	3.83	1.19E-03	2.03	3.86E-03
U043902	Anthocyanidin 3-O-glucosyltransferase 2 [Source:SWISS;ACC:Q40285]	0.48	4.63	3.79	6.52E-01	2.16	9.17E-02	0.05	6.06E-01
U016560	Defensin-like protein {ECO:0000250|UniProtKB:Q09198} [Source:SWISS;ACC:B5LZ79]	24.21	323.44	4.26	1.11E-02	1.96	1.36E-02	0.57	5.35E-02
U033935	Uncharacterized protein YnbD [Source:SWISS;ACC:P76093]	2.5	40.54	4.55	3.14E-02	3.15	3.79E-05	1.71	6.78E-03
U033988	Cytochrome P450 86B1 [Source:SWISS;ACC:Q9FMY1]	1.71	30.85	4.8	1.34E-04	3.96	1.47E-04	2.58	9.08E-04

Unigene ID	Description	Transcriptome analysis by NGS				qRT-PCR			
		RPKM in NW	RPKM in AD	Log_2FC	P-value	Sampled in 2014		Sampled in 2019	
						Log_2FC	P-value	Log_2FC	P-value
U024030	Copper transporter 6 [Source:SWISS;ACC:Q8GWP3]	3.3	104.57	5.52	1.29E-03	6.06	1.12E-05	2.68	4.24E-03
U014148	Probable aquaporin NIP-type [Source:SWISS;ACC:P49173]	0.82	31.55	5.76	1.30E-02	8.55	8.60E-05	4.66	8.94E-04
U020146	Copper transporter 5 [Source:SWISS;ACC:Q93VM8]	27.07	1769.93	6.51	1.41E-04	6.15	7.49E-05	2.54	7.84E-03
U026215	Caffeic acid 3-O-methyltransferase [Source:SWISS;ACC:P28002]	0.19	19.63	7.27	4.68E-03	8.39	3.49E-03	8.79	4.56E-03
U019322	Probable non-specific lipid-transfer protein AKCS9 [Source:SWISS;ACC:Q43681]	9.72	1705.88	7.94	3.13E-07	11.33	1.21E-02	7.32	4.26E-03
U031040	Protein TIFY 10A [Source:SWISS;ACC:Q9LMA8]	0.4	78.62	8.17	1.39E-04	3.59	3.53E-05	2.14	3.76E-05
U002745	Agglutinin [Source:SWISS;ACC:P82859]	2.51	860.89	8.98	5.05E-05	9.16	1.27E-02	5.52	1.24E-03
U000899	1-deoxy-D-xylulose-5-phosphate synthase 1, chloroplastic [Source:SWISS;ACC:O22567]	0	11.63	12.7	5.61E-02	10.26	1.50E-02	2.62	8.65E-03
U015169	Fatty-acid-binding protein 1 [Source:SWISS;ACC:Q9M1X2]	0	35.3	14.7	1.10E-03	6.88	9.19E-03	4.69	3.48E-04

Supplementary Table S3. Expression levels of differentially expressed genes.

Unigene ID	Length	Description	FPKM		Log_2FC	P-value	Gene ontology
			NW	AD			
U050360	1,273	-	253.62	0	-17.4	1.87E-07	-
U059230	1,413	-	186.56	0	-17.1	3.13E-07	-
U089491	2,172	-	95.77	0	-16.9	5.17E-07	-
U032395	1,903	Probable protein S-acyltransferase 7 [Source:SWISS;ACC:Q0WQK2]	98.3	0	-16.7	8.01E-07	GO:0005886, GO:0008270, GO:0016021, GO:0019706
U020637	3,121	-	51.41	0	-16.6	1.10E-06	-
U004151	1,023	Eukaryotic peptide chain release factor subunit 1-3 [Source:SWISS;ACC:P35614]	155.48	0	-16.2	2.64E-06	GO:0003747, GO:0005737, GO:0006415, GO:0016149
U052925	3,722	Elongation factor 2 [Source:SWISS;ACC:O23755]	33.2	0	-16.2	2.79E-06	GO:0003746, GO:0003924, GO:0005525, GO:0005737
U054922	2,803	Probable pectin methyltransferase QUA2 [Source:SWISS;ACC:Q9C9Q8]	43.05	0	-16.1	3.40E-06	GO:0000139, GO:0005768, GO:0005794, GO:0005802, GO:0007155, GO:0008168, GO:0009735, GO:0010289, GO:0016021, GO:0048364, GO:0048367
U064359	783	-	191.83	0	-16	5.33E-06	-

Unigene ID	Length	Description	FPKM		Log$_2$FC	P-value	Gene ontology
			NW	AD			
U033430	1,730	-	58.03	0	-15.8	9.39E-06	-
U036203	1,168	-	94.66	0	-15.8	9.34E-06	-
U026643	2,236	Putative pentatricopeptide repeat-containing protein At1g12700, mitochondrial [Source:SWISS;ACC:P0C7Q7]	41.88	0	-15.7	1.05E-05	GO:0005739
U033957	1,075	Peptidyl-prolyl cis-trans isomerase CYP20-2, chloroplastic [Source:SWISS;ACC:Q9ASS6]	97.79	0	-15.7	1.29E-05	GO:0003755, GO:0006457, GO:0009507, GO:0009533, GO:0009534, GO:0009535, GO:0009543, GO:0009579, GO:0010275, GO:0031969, GO:0031977, GO:0042277, GO:0043424
U044466	1,649	UDP-galactose transporter 2 [Source:SWISS;ACC:Q9SRE4]	57.84	0	-15.7	1.20E-05	GO:0005457, GO:0005459, GO:0005460, GO:0009624, GO:0015783, GO:0015786, GO:0016021, GO:0072334
U054924	2,793	Probable pectin methyltransferase QUA2 [Source:SWISS;ACC:Q9C9Q8]	30.29	0	-15.6	1.41E-05	GO:0000139, GO:0005768, GO:0005794, GO:0005802, GO:0007155, GO:0008168, GO:0009735, GO:0010289, GO:0016021, GO:0048364, GO:0048367
U100261	1,051	-	94.76	0	-15.6	1.65E-05	-
U014898	2,669	Dammarenediol II synthase [Source:SWISS;ACC:Q08IT1]	26.96	0	-15.4	2.79E-05	GO:0016021, GO:0016829, GO:0016866
U033823	636	Ubiquitin-60S ribosomal protein L40 [Source:SWISS;ACC:P51423]	175.36	0	-15.4	3.09E-05	GO:0003735, GO:0005634, GO:0005840, GO:0006412

Unigene ID	Length	Description	FPKM		Log_2FC	P-value	Gene ontology
			NW	AD			
U048120	870	Histone H2AX [Source:SWISS;ACC:P35063]	109.92	0	-15.4	2.68E-05	GO:0000786, GO:0003677, GO:0005634
U030774	501	Pentatricopeptide repeat-containing protein At2g25580 [Source:SWISS;ACC:Q680H3]	262.5	0	-15.3	4.09E-05	-
U091402	1,993	-	36.08	0	-15.3	3.30E-05	-
U020594	1,529	-	44.2	0	-15.2	5.23E-05	-
U021588	2,430	Exocyst complex component EXO70A1 [Source:SWISS;ACC:Q9LZD3]	26.04	0	-15.2	4.95E-05	GO:0000145, GO:0005576, GO:0005618, GO:0005829, GO:0005856, GO:0005886, GO:0006887, GO:0009524
U031677	2,132	(+)-larreatricin hydroxylase, chloroplastic [Source:SWISS;ACC:Q6UIL3]	30.99	0	-15.2	4.48E-05	GO:0004097, GO:0009543, GO:0046148, GO:0046872, GO:1901708, GO:1901709
U055255	867	Pleiotropic drug resistance protein 2 [Source:SWISS;ACC:Q2PCF1]	94.08	0	-15.2	5.16E-05	GO:0005524, GO:0006810, GO:0016021, GO:0016887
U017481	1,575	Enoyl-[acyl-carrier-protein] reductase [NADH], chloroplastic [Source:SWISS;ACC:Q9SLA8]	39.73	0	-15.1	6.93E-05	GO:0004318, GO:0005507, GO:0005835, GO:0006633, GO:0009507, GO:0009570, GO:0009579, GO:0009941, GO:0016631
U024689	1,773	Protein notum homolog [Source:SWISS;ACC:Q8R116]	34.36	0	-15.1	7.03E-05	GO:0005576, GO:0016787

Unigene ID	Length	Description	FPKM		Log_2FC	P-value	Gene ontology
			NW	AD			
U030016	1,731	UPF0481 protein At3g47200 [Source:SWISS;ACC:Q9SD53]	37.45	0	-15.1	5.56E-05	GO:0005886, GO:0016021
U025777	928	-	74.23	0	-15	9.08E-05	-
U036301	1,313	50S ribosomal protein L22 {ECO:0000255|HAMAP-Rule:MF_01331} [Source:SWISS;ACC:A6U864]	47.11	0	-15	8.67E-05	GO:0003735, GO:0006412, GO:0015934, GO:0019843
U036204	831	-	83.88	0	-14.9	1.06E-04	-
U042866	1,401	-	41.83	0	-14.9	1.01E-04	-
U050920	1,891	NEDD8-activating enzyme E1 regulatory subunit {ECO:0000305} [Source:SWISS;ACC:P42744]	27.49	0	-14.8	1.27E-04	GO:0000166, GO:0003824, GO:0005634, GO:0005829, GO:0006281, GO:0007131, GO:0009414, GO:0009734, GO:0009735, GO:0009965, GO:0010252, GO:0016567, GO:0045116
U051061	2,344	KH domain-containing protein At4g18375 [Source:SWISS;ACC:P58223]	21	0	-14.8	1.38E-04	GO:0003723, GO:0005634
U054928	2,403	Zinc finger CCCH domain-containing protein 19 [Source:SWISS;ACC:Q9SIV5]	20.2	0	-14.8	1.44E-04	GO:0003677, GO:0005634, GO:0006352, GO:0008270, GO:0010964, GO:0016570, GO:0031047, GO:0032776, GO:0042393

Unigene ID	Length	Description	FPKM		Log$_2$FC	P-value	Gene ontology
			NW	AD			
U013224	1,075	Probable receptor-like protein kinase At2g39360 [Source:SWISS;ACC:O80623]	51.02	0	-14.7	1.78E-04	GO:0004674, GO:0005524, GO:0005886, GO:0016021
U024932	1,065	-	51.29	0	-14.7	1.85E-04	-
U025917	1,049	-	53.3	0	-14.7	1.72E-04	-
U035855	985	Polyubiquitin [Source:SWISS;ACC:P0CG83]	56.1	0	-14.7	1.97E-04	GO:0005634, GO:0005737
U051690	1,583	-	31.03	0	-14.7	1.81E-04	-
U054710	554	-	143.07	0	-14.7	1.99E-04	-
U007390	1,263	-	38.98	0	-14.6	2.24E-04	-
U015372	676	-	95.41	0	-14.6	2.33E-04	-
U016920	938	-	55.86	0	-14.6	2.61E-04	-
U017856	817	-	69.22	0	-14.6	2.49E-04	-
U021134	1,418	-	32.23	0	-14.6	2.67E-04	-
U027596	840	Heavy metal-associated isoprenylated plant protein 26 [Source:SWISS;ACC:Q9SZN7]	64.32	0	-14.6	2.83E-04	GO:0005886, GO:0009506, GO:0010286, GO:0030001, GO:0031965, GO:0046872

Unigene ID	Length	Description	FPKM		Log_2FC	P-value	Gene ontology
			NW	AD			
U029501	1,069	Chalcone--flavonone isomerase [Source:SWISS;ACC:Q45QI7]	48.24	0	-14.6	2.29E-04	GO:0009813, GO:0045430
U038944	1,403	CBS domain-containing protein CBSX2, chloroplastic [Source:SWISS;ACC:Q9C5D0]	34.09	0	-14.6	2.24E-04	GO:0009507, GO:0009570, GO:0030554, GO:0045454
U046248	2,661	-	15.38	0	-14.6	2.69E-04	-
U049419	2,266	Ubiquitin carboxyl-terminal hydrolase 22 [Source:SWISS;ACC:Q9LEW0]	18.57	0	-14.6	2.61E-04	GO:0006511, GO:0008234, GO:0008270, GO:0036459
U050533	1,855	Beta-lactamase-like protein 2 [Source:SWISS;ACC:Q0V9A9]	24.47	0	-14.6	2.20E-04	GO:0016787, GO:0046872
U056373	1,952	Probable galacturonosyltransferase-like 7 [Source:SWISS;ACC:Q8VYF4]	22.67	0	-14.6	2.35E-04	GO:0000139, GO:0005794, GO:0016021, GO:0045489, GO:0047262, GO:0071555
U001007	651	-	95.01	0	-14.5	3.03E-04	-
U018210	1,675	26S proteasome non-ATPase regulatory subunit 4 homolog [Source:SWISS;ACC:P55034]	25.82	0	-14.5	2.86E-04	GO:0000502, GO:0001653, GO:0005634, GO:0005829, GO:0006974, GO:0007165, GO:0008540, GO:0009408, GO:0009555, GO:0009651, GO:0009733, GO:0009735, GO:0009737, GO:0009744, GO:0010029, GO:0010150, GO:0016020, GO:0030163, GO:0031593, GO:0043161, GO:0043248, GO:0048366, GO:0048455, GO:0048528, GO:0048767, GO:0051788

Unigene ID	Length	Description	FPKM		Log_2FC	P-value	Gene ontology
			NW	AD			
U021358	914	-	53.13	0	-14.5	3.65E-04	-
U032284	1,788	Glutathione S-transferase DHAR2 [Source:SWISS;ACC:Q9FRL8]	23.72	0	-14.5	2.94E-04	GO:0004364, GO:0005829, GO:0005886, GO:0009636, GO:0010731, GO:0043295, GO:0045174, GO:0055114
U034524	1,938	Acetolactate synthase small subunit 1, chloroplastic [Source:SWISS;ACC:Q9FFF4]	20.9	0	-14.5	3.32E-04	GO:0003984, GO:0005829, GO:0006551, GO:0006573, GO:0009097, GO:0009099, GO:0009507, GO:0016597
U038804	2,208	Protein NRT1/ PTR FAMILY 8.3 [Source:SWISS;ACC:P46032]	18.59	0	-14.5	2.91E-04	GO:0000325, GO:0005773, GO:0005774, GO:0005794, GO:0009705, GO:0015031, GO:0015197, GO:0015334, GO:0015833, GO:0016021, GO:0042936, GO:0042937, GO:0042938, GO:0042939
U038806	2,192	Protein NRT1/ PTR FAMILY 8.3 [Source:SWISS;ACC:P46032]	18.58	0	-14.5	3.03E-04	GO:0000325, GO:0005773, GO:0005774, GO:0005794, GO:0009705, GO:0015031, GO:0015197, GO:0015334, GO:0015833, GO:0016021, GO:0042936, GO:0042937, GO:0042938, GO:0042939
U042081	1,743	Putative receptor protein kinase ZmPK1 [Source:SWISS;ACC:P17801]	24.19	0	-14.5	3.06E-04	GO:0004674, GO:0005524, GO:0016021, GO:0048544
U054926	2,464	Zinc finger CCCH domain-containing protein 19 [Source:SWISS;ACC:Q9SIV5]	15.5	0	-14.5	3.65E-04	GO:0003677, GO:0005634, GO:0006352, GO:0008270, GO:0010964, GO:0016570, GO:0031047, GO:0032776, GO:0042393
U064350	1,028	-	45	0	-14.5	3.68E-04	-

Unigene ID	Length	Description	FPKM		Log_2FC	P-value	Gene ontology
			NW	AD			
U024593	1,385	Putative ALA-interacting subunit 2 [Source:SWISS;ACC:Q67YS6]	30.42	0	-14.4	3.72E-04	GO:0016021
U036164	1,075	-	39.66	0	-14.4	4.72E-04	-
U070452	687	-	79.94	0	-14.4	4.14E-04	-
U085823	544	-	122.98	0	-14.4	4.10E-04	-
U058839	1,121	Putative Holliday junction resolvase {ECO:0000255|HAMAP-Rule:MF_00651} [Source:SWISS;ACC:Q045P4]	35.31	0	-14.3	5.91E-04	GO:0003676, GO:0004518, GO:0005737, GO:0006281, GO:0006310
U094686	2,721	Serine/threonine-protein phosphatase 5 [Source:SWISS;ACC:Q84K11]	12.34	0	-14.3	5.64E-04	GO:0004721, GO:0005789, GO:0006470, GO:0016021, GO:0016607, GO:0031965, GO:0046872
U014751	2,528	Zinc finger CCCH domain-containing protein 19 [Source:SWISS;ACC:Q9SIV5]	12.38	0	-14.2	7.58E-04	GO:0003677, GO:0005634, GO:0006352, GO:0008270, GO:0010964, GO:0016570, GO:0031047, GO:0032776, GO:0042393
U015093	678	-	70.74	0	-14.2	7.05E-04	-
U033355	1,284	5'-adenylylsulfate reductase-like 5 [Source:SWISS;ACC:Q93YX4]	27.3	0	-14.2	7.95E-04	GO:0016021, GO:0045454
U056368	1,711	Probable galacturonosyltransferase-like 7 [Source:SWISS;ACC:Q8VYF4]	19.68	0	-14.2	7.22E-04	GO:0000139, GO:0005794, GO:0016021, GO:0045489, GO:0047262, GO:0071555

Unigene ID	Length	Description	FPKM		Log_2FC	P-value	Gene ontology
			NW	AD			
U001774	731	-	56.4	0	-14.1	1.02E-03	-
U023278	1,567	F-box/kelch-repeat protein At3g06240 [Source:SWISS;ACC:Q8GXC7]	21.06	0	-14.1	8.25E-04	-
U023546	1,215	-	28.89	0	-14.1	8.35E-04	-
U029606	1,290	-	25.24	0	-14.1	1.04E-03	-
U035277	2,224	DEAD-box ATP-dependent RNA helicase 53 [Source:SWISS;ACC:Q9LUW5]	13.64	0	-14.1	9.00E-04	GO:0000373, GO:0003723, GO:0004386, GO:0005524, GO:0005618, GO:0005730, GO:0005739, GO:0009409, GO:0043234
U040698	811	-	48.07	0	-14.1	1.02E-03	-
U042942	2,412	Peptide-N(4)-(N-acetyl-beta-glucosaminyl)asparagine amidase [Source:SWISS;ACC:Q9FGY9]	12.11	0	-14.1	9.96E-04	GO:0000224, GO:0003684, GO:0005634, GO:0005829, GO:0006289, GO:0009751, GO:0010188, GO:0010193, GO:0046872
U057327	1,254	DNA-damage-repair/toleration protein DRT100 [Source:SWISS;ACC:Q00874]	26.44	0	-14.1	9.96E-04	GO:0000166, GO:0006281, GO:0009411, GO:0009507, GO:0042493
U058119	980	-	37.37	0	-14.1	9.35E-04	-
U062215	730	-	58.45	0	-14.1	9.11E-04	-
U063934	976	-	37.77	0	-14.1	9.23E-04	-

Unigene ID	Length	Description	FPKM		Log_2FC	P-value	Gene ontology
			NW	AD			
U085186	1,036	-	34.01	0	-14.1	9.96E-04	-
U012721	1,041	Protein CREG1 [Source:SWISS;ACC:O75629]	32.98	0	-14	1.09E-03	GO:0003714, GO:0005667, GO:0006357, GO:0007275, GO:0008283, GO:0010181, GO:0016491, GO:0040008, GO:0070062, GO:1903507
U019937	1,192	Probable calcium-binding protein CML21 [Source:SWISS;ACC:Q52K82]	26.12	0	-14	1.32E-03	GO:0005509, GO:0005737
U021458	1,032	-	33.81	0	-14	1.04E-03	-
U024736	1,320	Quinone oxidoreductase 1 [Source:SWISS;ACC:P28304]	23.43	0	-14	1.21E-03	GO:0003960, GO:0005737, GO:0008270
U028119	620	-	73.36	0	-14	1.09E-03	-
U035810	841	-	44.38	0	-14	1.11E-03	-
U040374	1,673	Probable membrane-associated 30 kDa protein, chloroplastic [Source:SWISS;ACC:Q8S0J7]	18	0	-14	1.09E-03	GO:0009508, GO:0009535, GO:0009570, GO:0009706, GO:0010027, GO:0016032, GO:0016050
U049119	818	-	45.81	0	-14	1.15E-03	-
U056412	1,167	-	27.47	0	-14	1.21E-03	-
U063380	1,141	-	28.26	0	-14	1.21E-03	-

Unigene ID	Length	Description	FPKM		Log_2FC	P-value	Gene ontology
			NW	AD			
U017683	1,906	Pyruvate kinase, cytosolic isozyme [Source:SWISS;ACC:Q42806]	14.48	0	-13.9	1.37E-03	GO:0000287, GO:0004743, GO:0005524, GO:0005737, GO:0006096, GO:0030955
U025727	1,160	26S proteasome non-ATPase regulatory subunit 4 homolog [Source:SWISS;ACC:P55034]	25.81	0	-13.9	1.56E-03	GO:0000502, GO:0001653, GO:0005634, GO:0005829, GO:0006974, GO:0007165, GO:0008540, GO:0009408, GO:0009555, GO:0009651, GO:0009733, GO:0009735, GO:0009737, GO:0009744, GO:0010029, GO:0010150, GO:0016020, GO:0030163, GO:0031593, GO:0043161, GO:0043248, GO:0048366, GO:0048455, GO:0048528, GO:0048767, GO:0051788
U041818	2,015	Multidrug and toxin extrusion protein 1 [Source:SWISS;ACC:A4IIS8]	12.97	0	-13.9	1.60E-03	GO:0005886, GO:0015238, GO:0015297, GO:0016021
U047571	1,398	-	20.33	0	-13.9	1.53E-03	-
U089879	795	-	44.49	0	-13.9	1.49E-03	-
U093294	672	-	59	0	-13.9	1.43E-03	-
U011831	1,651	Putative ribonuclease H protein At1g65750 [Source:SWISS;ACC:P0C2F6]	15.61	0	-13.8	1.91E-03	GO:0003676, GO:0004523, GO:0046872
U013900	592	-	67.87	0	-13.8	1.94E-03	-
U014897	2,131	Dammarenediol II synthase [Source:SWISS;ACC:Q08IT1]	11.32	0	-13.8	2.06E-03	GO:0016021, GO:0016829, GO:0016866

Unigene ID	Length	Description	FPKM		Log_2FC	P-value	Gene ontology
			NW	AD			
U020608	2,242	-	11.2	0	-13.8	1.75E-03	-
U020780	1,725	Structural maintenance of chromosomes protein 1 [Source:SWISS;ACC:Q6Q1P4]	14.94	0	-13.8	1.86E-03	GO:0003682, GO:0005524, GO:0005634, GO:0006281, GO:0007059, GO:0007064, GO:0008278, GO:0051321
U024732	1,718	Pyruvate dehydrogenase E1 component subunit beta-1, mitochondrial [Source:SWISS;ACC:Q38799]	14.67	0	-13.8	2.00E-03	GO:0004739, GO:0005730, GO:0005739, GO:0005759, GO:0005774, GO:0006086, GO:0006096, GO:0042742, GO:0048046
U028329	1,258	Uncharacterized protein ycf37 [Source:SWISS;ACC:O78458]	22.34	0	-13.8	1.77E-03	GO:0009507
U028338	1,325	-	20.89	0	-13.8	1.77E-03	-
U034171	602	-	65.71	0	-13.8	1.94E-03	-
U040774	751	-	44	0	-13.8	2.10E-03	-
U043406	647	-	56.41	0	-13.8	2.10E-03	-
U048417	2,684	Serine/threonine protein phosphatase 2A regulatory subunit B"beta [Source:SWISS;ACC:Q5QIT3]	8.74	0	-13.8	2.03E-03	GO:0005509, GO:0005829
U051216	3,261	Serine/threonine-protein kinase EDR1 [Source:SWISS;ACC:Q9FPR3]	7.18	0	-13.8	1.91E-03	GO:0000165, GO:0000186, GO:0002229, GO:0004709, GO:0004712, GO:0005524, GO:0005634, GO:0005769, GO:0005783, GO:0005802, GO:0005829, GO:0008219,

Unigene ID	Length	Description	FPKM		Log$_2$FC	P-value	Gene ontology
			NW	AD			
							GO:0009414, GO:0009617, GO:0009620, GO:0009723, GO:0009738, GO:0009788, GO:0009873, GO:0016301, GO:0046777, GO:1900150, GO:1900424, GO:2000031
U053082	660	-	56	0	-13.8	1.91E-03	-
U054349	2,017	Protein MOR1 [Source:SWISS;ACC:Q5N749]	12.51	0	-13.8	1.80E-03	GO:0005737, GO:0005874
U000600	958	Putative Holliday junction resolvase {ECO:0000255|HAMAP-Rule:MF_00651} [Source:SWISS;ACC:B8I4S7]	29.11	0	-13.7	2.54E-03	GO:0003676, GO:0004518, GO:0005737, GO:0006281, GO:0006310
U003895	1,119	-	24.27	0	-13.7	2.27E-03	-
U018328	1,323	-	19.81	0	-13.7	2.16E-03	-
U032304	2,311	Molybdopterin biosynthesis protein CNX1 [Source:SWISS;ACC:Q39054]	9.82	0	-13.7	2.46E-03	GO:0005524, GO:0005829, GO:0006777, GO:0009734, GO:0030151, GO:0061598, GO:0061599
U041414	1,506	Protein NLP3 [Source:SWISS;ACC:Q5NB82]	16.38	0	-13.7	2.38E-03	GO:0003677, GO:0005634, GO:0006351, GO:0006355
U043316	1,777	Polyadenylate-binding protein RBP47B' [Source:SWISS;ACC:Q8VXZ9]	13.48	0	-13.7	2.34E-03	GO:0000166, GO:0005634, GO:0006397, GO:0008143, GO:0010494, GO:0034605

Unigene ID	Length	Description	FPKM		Log_2FC	P-value	Gene ontology
			NW	AD			
U043740	1,432	Probable serine/threonine-protein kinase DDB_G0276461 [Source:SWISS;ACC:Q86HW6]	17.61	0	-13.7	2.30E-03	GO:0004674, GO:0005524
U046340	1,973	Probable polyamine oxidase 5 [Source:SWISS;ACC:Q9SU79]	12.16	0	-13.7	2.20E-03	GO:0016491
U046869	1,478	F-box/FBD/LRR-repeat protein At1g13570 [Source:SWISS;ACC:Q9FZ70]	16.78	0	-13.7	2.38E-03	-
U048221	1,033	11-beta-hydroxysteroid dehydrogenase 1B [Source:SWISS;ACC:P0DKC6]	26.57	0	-13.7	2.42E-03	GO:0005811, GO:0006694, GO:0016021, GO:0070524
U054341	2,348	Glutamate receptor 3.6 [Source:SWISS;ACC:Q84W41]	9.58	0	-13.7	2.50E-03	GO:0004970, GO:0005234, GO:0005262, GO:0005886, GO:0006816, GO:0008066, GO:0009611, GO:0009864, GO:0016021, GO:0019722, GO:0070588, GO:0071230
U080883	676	-	51.66	0	-13.7	2.20E-03	-
U004461	1,315	-	18.42	0	-13.6	2.85E-03	-
U014436	1,914	Cyclin-A2-1 [Source:SWISS;ACC:Q39071]	11.52	0	-13.6	3.00E-03	GO:0000079, GO:0005634, GO:0007049, GO:0051301
U022553	911	14 kDa zinc-binding protein [Source:SWISS;ACC:P42856]	29.47	0	-13.6	3.11E-03	GO:0003824

Unigene ID	Length	Description	FPKM		Log_2FC	P-value	Gene ontology
			NW	AD			
U023192	983	-	26.31	0	-13.6	3.17E-03	-
U027613	866	ER membrane protein complex subunit 4 [Source:SWISS;ACC:Q6P011]	32.78	0	-13.6	2.76E-03	GO:0016021, GO:0072546
U033714	1,624	Polycomb group protein FERTILIZATION-INDEPENDENT ENDOSPERM [Source:SWISS;ACC:Q9LT47]	13.69	0	-13.6	3.22E-03	GO:0003700, GO:0005677, GO:0006349, GO:0006351, GO:0007275, GO:0009409, GO:0016571, GO:0043078, GO:2000014
U035644	1,172	-	20.93	0	-13.6	3.06E-03	-
U040546	2,671	Gamma-tubulin complex component 2 [Source:SWISS;ACC:Q9C5H9]	7.57	0	-13.6	3.46E-03	GO:0000226, GO:0000922, GO:0000930, GO:0005635, GO:0005737, GO:0005938, GO:0033566, GO:0048229, GO:0055028, GO:0090063
U041696	1,120	Glucuronoxylan 4-O-methyltransferase 3 [Source:SWISS;ACC:Q9LQ32]	22.13	0	-13.6	3.11E-03	GO:0000139, GO:0005794, GO:0005976, GO:0009808, GO:0016021, GO:0030775, GO:0045491
U041844	1,920	-	11.06	0	-13.6	3.40E-03	-
U043803	1,172	-	21.39	0	-13.6	2.85E-03	-
U046469	2,221	Ribulose-1,5 bisphosphate carboxylase/oxygenase large subunit N-methyltransferase, chloroplastic [Source:SWISS;ACC:P94026]	9.66	0	-13.6	3.06E-03	GO:0009507, GO:0030785

Unigene ID	Length	Description	FPKM		Log_2FC	P-value	Gene ontology
			NW	AD			
U084805	518	-	75.68	0	-13.6	3.40E-03	-
U093736	889	-	31.1	0	-13.6	2.90E-03	-
U023279	925	-	26.83	0	-13.5	4.00E-03	-
U024678	1,351	Caffeic acid 3-O-methyltransferase [Source:SWISS;ACC:Q43239]	16.69	0	-13.5	3.59E-03	GO:0009809, GO:0047763
U024733	1,636	Pyruvate dehydrogenase E1 component subunit beta-1, mitochondrial [Source:SWISS;ACC:Q38799]	12.73	0	-13.5	4.08E-03	GO:0004739, GO:0005730, GO:0005739, GO:0005759, GO:0005774, GO:0006086, GO:0006096, GO:0042742, GO:0048046
U028030	806	-	33.34	0	-13.5	3.79E-03	-
U039931	2,635	Protease 2 [Source:SWISS;ACC:Q59536]	7.64	0	-13.5	3.52E-03	GO:0004252, GO:0070008
U040221	2,279	F-box/FBD/LRR-repeat protein At1g13570 [Source:SWISS;ACC:Q9FZ70]	8.72	0	-13.5	3.93E-03	-
U041487	1,245	Probable mediator of RNA polymerase II transcription subunit 36b [Source:SWISS;ACC:Q9FEF8]	18.62	0	-13.5	3.52E-03	GO:0001510, GO:0005730, GO:0006351, GO:0006355, GO:0006364, GO:0008033, GO:0008168, GO:0016020, GO:0030515, GO:0030529
U041904	1,402	MLO-like protein 5 [Source:SWISS;ACC:O22815]	15.27	0	-13.5	4.16E-03	GO:0006952, GO:0009607, GO:0016021

Unigene ID	Length	Description	FPKM		Log_2FC	P-value	Gene ontology
			NW	AD			
U042156	1,042	-	22.29	0	-13.5	4.24E-03	-
U043033	2,778	Protein MEI2-like 2 [Source:SWISS;ACC:Q6ZI17]	7.14	0	-13.5	3.65E-03	GO:0000166, GO:0003723
U046871	630	-	51.19	0	-13.5	3.46E-03	-
U048740	2,749	-	7.03	0	-13.5	4.00E-03	-
U053523	1,242	Xylosyltransferase 2 [Source:SWISS;ACC:Q9EPL0]	18.3	0	-13.5	3.79E-03	GO:0000139, GO:0005789, GO:0006024, GO:0008375, GO:0015012, GO:0016021, GO:0030158, GO:0030166, GO:0030206, GO:0030210
U087535	764	-	35.37	0	-13.5	4.08E-03	-
U004138	684	-	41.06	0	-13.4	4.57E-03	-
U006160	647	-	44.4	0	-13.4	4.84E-03	-
U043269	1,232	Tubby-like F-box protein 5 [Source:SWISS;ACC:Q6Z2G9]	17.18	0	-13.4	4.84E-03	-
U029545	1,731	-	187.31	0.07	-10.8	5.52E-07	-
U012666	980	-	347.44	0.14	-10.7	7.43E-07	-
U089492	2,152	-	80.45	0.05	-9.97	3.49E-06	-

Unigene ID	Length	Description	FPKM		Log_2FC	P-value	Gene ontology
			NW	AD			
U016070	1,001	-	363.21	0.28	-9.77	1.40E-06	-
U008940	10,515	Retrovirus-related Pol polyprotein from transposon TNT 1-94 [Source:SWISS;ACC:P10978]	15.37	0.02	-9.03	6.85E-06	GO:0003676, GO:0003964, GO:0004190, GO:0004519, GO:0008270, GO:0015074
U029524	1,927	Origin of replication complex subunit 5 {ECO:0000303|PubMed:16179646} [Source:SWISS;ACC:Q6EWX0]	46.19	0.06	-8.98	4.65E-05	GO:0000808, GO:0005634, GO:0006260
U038860	3,257	Arginine--tRNA ligase {ECO:0000255|HAMAP-Rule:MF_00123} [Source:SWISS;ACC:Q5N643]	245.41	0.32	-8.94	4.67E-06	GO:0004814, GO:0005524, GO:0005737, GO:0006420
U006456	615	-	217.03	0.32	-8.93	5.41E-05	-
U093336	541	-	195.2	0.41	-8.42	2.16E-04	-
U046195	1,738	-	427.2	0.89	-8.28	9.48E-06	-
U061788	838	-	78.12	0.18	-8.18	4.01E-04	-
U091458	748	-	90.55	0.22	-8.15	4.45E-04	-
U050685	735	Probable V-type proton ATPase subunit H [Source:SWISS;ACC:Q84ZC0]	185.26	0.47	-8.09	6.61E-05	GO:0000221, GO:0000325, GO:0000902, GO:0005794, GO:0005886, GO:0006486, GO:0006623, GO:0006816, GO:0007030, GO:0007033, GO:0009507, GO:0009651,

Unigene ID	Length	Description	FPKM		Log_2FC	P-value	Gene ontology
			NW	AD			
							GO:0015991, GO:0016049, GO:0046961, GO:0048193
U028564	792	-	78.33	0.2	-8.04	5.49E-04	-
U025729	1,229	26S proteasome non-ATPase regulatory subunit 4 homolog [Source:SWISS;ACC:P55034]	99.33	0.27	-7.94	7.83E-05	GO:0000502, GO:0001653, GO:0005634, GO:0005829, GO:0006974, GO:0007165, GO:0008540, GO:0009408, GO:0009555, GO:0009651, GO:0009733, GO:0009735, GO:0009737, GO:0009744, GO:0010029, GO:0010150, GO:0016020, GO:0030163, GO:0031593, GO:0043161, GO:0043248, GO:0048366, GO:0048455, GO:0048528, GO:0048767, GO:0051788
U032373	1,214	Bidirectional sugar transporter SWEET10 [Source:SWISS;ACC:Q9LUE3]	38.34	0.11	-7.9	8.38E-04	GO:0005887, GO:0008515, GO:0015770, GO:0034219, GO:0051119
U031351	778	-	745.11	2.26	-7.82	1.51E-05	-
U031347	525	Glutamine synthetase nodule isozyme [Source:SWISS;ACC:P08282]	272.66	0.88	-7.81	1.58E-04	GO:0004356, GO:0005524, GO:0005737, GO:0006542, GO:0009399
U020461	838	-	59.08	0.18	-7.78	1.12E-03	-
U011807	1,365	Histone deacetylase 14 [Source:SWISS;ACC:Q941D6]	29.35	0.09	-7.73	1.28E-03	GO:0005634, GO:0005829, GO:0006351, GO:0006355, GO:0009507, GO:0032041, GO:0042903, GO:0043014, GO:0043621, GO:0046969, GO:0046970, GO:0048487, GO:0051721, GO:0090042, GO:0097372

Unigene ID	Length	Description	FPKM		Log_2FC	P-value	Gene ontology
			NW	AD			
U002412	764	-	128.12	0.43	-7.69	2.14E-04	-
U032319	981	-	84.04	0.29	-7.62	2.62E-04	-
U025313	2,098	Aspartic proteinase-like protein 2 [Source:SWISS;ACC:Q9S9K4]	82.14	0.28	-7.58	3.70E-05	GO:0004190, GO:0005886, GO:0031225, GO:0046658
U036718	801	-	51.16	0.2	-7.48	2.34E-03	-
U024671	781	-	53.01	0.2	-7.47	2.37E-03	-
U055468	2,777	-	31.82	0.12	-7.46	1.49E-04	-
U033468	568	-	89.66	0.37	-7.44	2.57E-03	-
U025164	979	-	77.31	0.31	-7.4	3.61E-04	-
U023749	1,518	Omega-6 fatty acid desaturase, endoplasmic reticulum [Source:SWISS;ACC:Q39287]	148.34	0.63	-7.26	4.83E-05	GO:0005789, GO:0006636, GO:0016021, GO:0016491
U040767	1,535	Endoglucanase 24 [Source:SWISS;ACC:Q93YQ7]	68.04	0.29	-7.23	1.80E-04	GO:0005576, GO:0008810, GO:0030245, GO:0071555
U054125	764	-	90.18	0.43	-7.19	7.92E-04	-
U094550	677	-	55.11	0.26	-7.19	4.50E-03	-

Unigene ID	Length	Description	FPKM		Log_2FC	P-value	Gene ontology
			NW	AD			
U058205	2,240	Nuclear pore complex protein NUP107 {ECO:0000303|PubMed:21189294} [Source:SWISS;ACC:Q8L748]	19.22	0.09	-7.09	1.37E-03	GO:0005635, GO:0005643, GO:0006810
U040819	1,650	DEAD-box ATP-dependent RNA helicase 46 [Source:SWISS;ACC:Q9LYJ9]	156.12	0.78	-7.02	6.10E-05	GO:0003723, GO:0005524, GO:0008026
U014899	2,578	Dammarenediol II synthase [Source:SWISS;ACC:Q08IT1]	16.67	0.08	-6.99	1.29E-03	GO:0016021, GO:0016829, GO:0016866
U040893	718	-	253.03	1.42	-6.95	1.43E-04	-
U015618	1,916	Cyclin-A2-1 [Source:SWISS;ACC:Q39071]	33.42	0.18	-6.92	5.94E-04	GO:0000079, GO:0005634, GO:0007049, GO:0051301
U027385	1,021	F-box protein GID2 [Source:SWISS;ACC:Q9STX3]	153.69	0.85	-6.92	1.31E-04	GO:0005634, GO:0009740, GO:0009845, GO:0010162, GO:0016567, GO:0019005
U036450	1,631	Malonyl-coenzyme A:anthocyanin 3-O-glucoside-6"-O-malonyltransferase {ECO:0000303|PubMed:12481098} [Source:SWISS;ACC:Q8GSN8]	41.36	0.23	-6.86	5.49E-04	GO:0009698, GO:0033809
U061875	1,171	Secoisolariciresinol dehydrogenase [Source:SWISS;ACC:Q94KL8]	238.94	1.45	-6.77	8.97E-05	GO:0000166, GO:0016491
U030015	1,698	Putative UPF0481 protein At3g02645 [Source:SWISS;ACC:P0C897]	23.24	0.14	-6.76	2.16E-03	GO:0016021

Unigene ID	Length	Description	FPKM		Log_2FC	P-value	Gene ontology
			NW	AD			
U023082	1,295	50S ribosomal protein L17 {ECO:0000255|HAMAP-Rule:MF_01368} [Source:SWISS;ACC:A0L5Z9]	67.84	0.48	-6.54	5.66E-04	GO:0003735, GO:0005840, GO:0006412
U012620	594	Cytochrome c oxidase subunit 6a, mitochondrial [Source:SWISS;ACC:Q9U070]	265.8	2.08	-6.5	3.85E-04	GO:0004129, GO:0005739, GO:0005751
U061943	965	Acid phosphatase 1 [Source:SWISS;ACC:P27061]	501.33	4.11	-6.35	1.59E-04	GO:0003993
U003068	643	Cysteine proteinase inhibitor 6 [Source:SWISS;ACC:Q8H0X6]	166.43	1.45	-6.34	8.84E-04	GO:0004869, GO:0005576, GO:0005783, GO:0005829, GO:0006952, GO:0006972, GO:0006979, GO:0009409, GO:0009414, GO:0010951, GO:0050897
U030941	1,760	CRAL-TRIO domain-containing protein C23B6.04c [Source:SWISS;ACC:Q9UU99]	21.9	0.2	-6.17	3.65E-03	GO:0005886, GO:0006696, GO:0008526, GO:0015914
U040169	1,710	Uncharacterized acetyltransferase At3g50280 [Source:SWISS;ACC:Q9SND9]	36.97	0.36	-6.05	1.43E-03	GO:0016747
U022274	1,490	Gamma carbonic anhydrase 2, mitochondrial [Source:SWISS;ACC:Q9C6B3]	71.81	0.71	-6.04	7.36E-04	GO:0004089, GO:0005739, GO:0005747, GO:0009507, GO:0009651, GO:0009853, GO:0009901, GO:0016020, GO:0031966, GO:0042802, GO:0045271, GO:0046872, GO:0070207, GO:2000377

Unigene ID	Length	Description	FPKM		Log_2FC	P-value	Gene ontology
			NW	AD			
U000039	1,728	3-ketoacyl-CoA synthase 17 [Source:SWISS;ACC:Q5XEP9]	60.37	0.61	-6.01	7.20E-04	GO:0006633, GO:0006970, GO:0009611, GO:0009922, GO:0010345, GO:0016021
U025273	2,014	Importin subunit alpha-1a [Source:SWISS;ACC:Q71VM4]	25.71	0.26	-6.01	2.57E-03	GO:0005634, GO:0006606, GO:0008565, GO:0016032, GO:0048471
U015711	674	Basic blue protein [Source:SWISS;ACC:P00303]	140.33	1.59	-5.95	1.63E-03	GO:0005507, GO:0009055, GO:0055114
U022396	1,820	Pentatricopeptide repeat-containing protein At1g62670, mitochondrial [Source:SWISS;ACC:Q9SXD1]	78.33	0.82	-5.95	5.24E-04	GO:0000966, GO:0003723, GO:0005739
U021839	2,000	Serine carboxypeptidase-like 16 [Source:SWISS;ACC:Q9C7D4]	383.02	4.29	-5.85	5.29E-04	GO:0004185, GO:0005576
U008278	1,043	Mini zinc finger protein 2 [Source:SWISS;ACC:Q9LJW5]	140.23	1.83	-5.67	8.99E-04	GO:0003677, GO:0005634, GO:0005737, GO:0006351, GO:0006355, GO:0007275, GO:0042803, GO:0046872
U025275	2,115	Importin subunit alpha-1a [Source:SWISS;ACC:Q71VM4]	159.49	2.1	-5.61	5.55E-04	GO:0005634, GO:0006606, GO:0008565, GO:0016032, GO:0048471
U043660	1,775	-	33.12	0.51	-5.39	3.87E-03	-
U091542	614	-	130.05	2.22	-5.38	4.62E-03	-
U054347	2,353	Protein MOR1 [Source:SWISS;ACC:Q5N749]	54.96	0.88	-5.32	1.43E-03	GO:0005737, GO:0005874

Unigene ID	Length	Description	FPKM		Log_2FC	P-value	Gene ontology
			NW	AD			
U020751	1,932	Flavonoid 3'-monooxygenase [Source:SWISS;ACC:Q9SBQ9]	132.27	2.64	-5.01	1.56E-03	GO:0005506, GO:0005789, GO:0009813, GO:0016021, GO:0016711, GO:0020037
U020721	2,948	ABC transporter G family member 39 [Source:SWISS;ACC:Q7PC84]	23.68	0.48	-4.98	4.24E-03	GO:0000302, GO:0005524, GO:0005886, GO:0006855, GO:0016021, GO:0016887
U041526	1,024	Bifunctional epoxide hydrolase 2 [Source:SWISS;ACC:P34913]	180.31	3.89	-4.95	2.20E-03	GO:0000287, GO:0004301, GO:0005102, GO:0005777, GO:0005829, GO:0006805, GO:0006874, GO:0006954, GO:0008217, GO:0009636, GO:0010628, GO:0015643, GO:0016311, GO:0016791, GO:0017144, GO:0019369, GO:0019373, GO:0033885, GO:0042577, GO:0042632, GO:0042803, GO:0044281, GO:0045909, GO:0046272, GO:0046839, GO:0070062, GO:0072593, GO:0090181
U050035	2,290	Putative 12-oxophytodienoate reductase 11 [Source:SWISS;ACC:B9FSC8]	55.52	1.16	-4.95	2.53E-03	GO:0010181, GO:0016491, GO:0031408
U025082	2,397	-	54.98	1.15	-4.94	2.43E-03	-
U056322	4,353	Auxin response factor 5 [Source:SWISS;ACC:Q6Z2W3]	20.66	0.45	-4.87	3.67E-03	GO:0003677, GO:0005634, GO:0006351, GO:0006355, GO:0009734
U051204	2,443	Subtilisin-like protease SDD1 {ECO:0000303|PubMed:10809670} [Source:SWISS;ACC:O64495]	45.77	1.03	-4.84	3.31E-03	GO:0004252, GO:0005618, GO:0008152, GO:0009897, GO:0010103, GO:0042127, GO:0048046

Unigene ID	Length	Description	FPKM		Log_2FC	P-value	Gene ontology
			NW	AD			
U020331	2,031	UDP-glycosyltransferase 76C1 [Source:SWISS;ACC:Q9FI99]	173.53	4.04	-4.79	2.19E-03	GO:0047807, GO:0080062
U030165	1,794	Protein TRIGALACTOSYLDIACYLGLYCEROL 4, chloroplastic [Source:SWISS;ACC:Q9M903]	57.95	1.36	-4.79	3.95E-03	GO:0005783, GO:0009507, GO:0009536, GO:0009707, GO:0009941, GO:0016020, GO:0016021, GO:0034196, GO:0042803, GO:0070300, GO:1990052
U050362	1,604	F-box protein At2g21930 [Source:SWISS;ACC:Q9SJ06]	80.77	2.07	-4.67	4.00E-03	-
U038233	1,141	Ethylene-responsive transcription factor RAP2-3 [Source:SWISS;ACC:P42736]	118.41	3.23	-4.6	4.59E-03	GO:0003677, GO:0003700, GO:0005634, GO:0005737, GO:0005886, GO:0006351, GO:0008219, GO:0009723, GO:0009735, GO:0009753, GO:0009873, GO:0010286, GO:0045893, GO:0051707
U059241	1,338	GDSL esterase/lipase At2g30310 [Source:SWISS;ACC:O22927]	143.7	3.91	-4.59	3.58E-03	GO:0005576, GO:0016042, GO:0016788
U036207	1,292	F-box/kelch-repeat protein At3g06240 [Source:SWISS;ACC:Q8GXC7]	160.56	4.97	-4.41	4.65E-03	-
U057020	3,430	BAG family molecular chaperone regulator 6 [Source:SWISS;ACC:O82345]	2	30.36	4.57	3.67E-03	GO:0005516, GO:0009408, GO:0009506, GO:0009644, GO:0010228, GO:0012502, GO:0042542, GO:0050832
U026287	830	Probable calcium-binding protein CML49 [Source:SWISS;ACC:Q8W4L0]	8.93	145.85	4.58	4.06E-03	GO:0005509

Unigene ID	Length	Description	FPKM		Log_2FC	P-value	Gene ontology
			NW	AD			
U033988	1,798	Cytochrome P450 86B1 [Source:SWISS;ACC:Q9FMY1]	1.71	30.85	4.8	4.54E-03	GO:0004497, GO:0005506, GO:0005783, GO:0005789, GO:0010345, GO:0016021, GO:0016705, GO:0020037, GO:0042761, GO:0071555
U034715	565	Osmotin-like protein [Source:SWISS;ACC:Q41350]	8.06	169.37	4.87	4.38E-03	GO:0000911, GO:0005576, GO:0009505, GO:0009506, GO:0009664, GO:0042545
U020714	638	Non-specific lipid-transfer protein [Source:SWISS;ACC:O04004]	40.51	854.81	4.91	1.99E-03	GO:0006869, GO:0008289
U030567	719	Glutaredoxin-C6 [Source:SWISS;ACC:Q8L9S3]	13.95	299.58	4.95	1.74E-03	GO:0005737, GO:0009055, GO:0015035, GO:0045454
U012168	599	-	6.99	157.92	4.99	3.46E-03	-
U010258	506	-	7.89	185.52	5.02	4.11E-03	-
U025332	1,401	-	0.91	23.76	5.32	4.88E-03	-
U024030	755	Copper transporter 6 [Source:SWISS;ACC:Q8GWP3]	3.3	104.57	5.52	1.65E-03	GO:0005375, GO:0005773, GO:0016021
U020931	1,701	Cytochrome P450 71A1 [Source:SWISS;ACC:P24465]	1.11	33.98	5.56	1.51E-03	GO:0004497, GO:0005506, GO:0005789, GO:0009835, GO:0016021, GO:0016705, GO:0020037
U015695	645	-	1.77	62.46	5.65	4.88E-03	-
U021974	762	-	1.34	48.13	5.71	4.34E-03	-

Unigene ID	Length	Description	FPKM		Log_2FC	P-value	Gene ontology
			NW	AD			
U027717	1,248	1-aminocyclopropane-1-carboxylate oxidase [Source:SWISS;ACC:A6BM06]	0.72	26.92	5.84	2.55E-03	GO:0005506, GO:0009693, GO:0009815, GO:0016706
U022675	763	-	2.48	99.81	5.87	1.07E-03	-
U019712	589	-	1.49	63.95	5.92	4.97E-03	-
U039580	1,146	-	0.82	32.86	5.92	2.38E-03	-
U034731	940	-	0.69	28.39	5.94	4.97E-03	-
U052726	575	-	9.66	440.43	6	3.18E-04	-
U014030	502	-	11.14	564.71	6.13	2.65E-04	-
U035174	650	Squamosa promoter-binding-like protein 12 [Source:SWISS;ACC:Q9S7P5]	4.95	253.45	6.19	3.01E-04	GO:0003677, GO:0003700, GO:0005634, GO:0006351, GO:0006355, GO:0046872
U062665	902	-	1.34	66.99	6.21	8.30E-04	-
U040895	858	-	0.67	35.34	6.28	2.86E-03	-
U019619	1,259	G-type lectin S-receptor-like serine/threonine-protein kinase RLK1 [Source:SWISS;ACC:Q39202]	0.38	19.57	6.3	3.22E-03	GO:0004674, GO:0005516, GO:0005524, GO:0005886, GO:0016021, GO:0030246

Unigene ID	Length	Description	FPKM		Log_2FC	P-value	Gene ontology
			NW	AD			
U082064	810	-	3.14	179.15	6.38	2.09E-04	-
U037951	542	Non-functional NADPH-dependent codeinone reductase 2 [Source:SWISS;ACC:Q9SQ64]	1.27	76.89	6.4	3.58E-03	GO:0005829, GO:0016491
U090030	1,085	-	0.34	19.4	6.4	4.56E-03	-
U021945	616	-	0.84	51.47	6.43	3.95E-03	-
U029679	888	-	1.77	103.34	6.43	3.42E-04	-
U014619	1,002	-	0.48	28.25	6.44	2.77E-03	-
U017338	535	-	10.8	686.74	6.46	1.29E-04	-
U032087	1,698	WAT1-related protein At4g19185 [Source:SWISS;ACC:Q8W4R9]	0.43	25.33	6.5	7.96E-04	GO:0005794, GO:0016021
U003618	538	-	1.42	93.2	6.51	1.97E-03	-
U056309	1,141	-	0.54	38.92	6.77	6.28E-04	-
U002410	933	-	9.06	677.19	6.8	1.10E-04	-
U019152	661	F-box/kelch-repeat protein At1g26930 [Source:SWISS;ACC:Q84M94]	0.72	56.32	6.81	1.85E-03	GO:0005634, GO:0005777

Unigene ID	Length	Description	FPKM		Log$_2$FC	P-value	Gene ontology
			NW	AD			
U035082	816	Transcription factor ORG3 [Source:SWISS;ACC:Q9M1K0]	0.69	55.04	6.87	7.89E-04	GO:0003677, GO:0003700, GO:0005634, GO:0006351, GO:0006355, GO:0010106, GO:0055072
U014292	705	-	4.77	397.23	6.91	6.16E-05	-
U055211	851	Miraculin [Source:SWISS;ACC:P13087]	0.32	27.43	6.97	3.16E-03	GO:0004866
U037831	720	-	0.5	48.28	7.12	1.16E-03	-
U018264	1,068	-	0.27	26.34	7.17	1.10E-03	-
U028470	647	-	1	107.6	7.25	2.93E-04	-
U026215	1,237	Caffeic acid 3-O-methyltransferase [Source:SWISS;ACC:P28002]	0.19	19.63	7.27	1.53E-03	GO:0009809, GO:0047763
U055418	507	-	1.31	148.4	7.28	3.94E-04	-
U083315	598	Non-specific lipid-transfer protein [Source:SWISS;ACC:O04004]	2.31	265.04	7.33	6.17E-05	GO:0006869, GO:0008289
U099116	633	-	8.87	1067.21	7.42	3.23E-05	-
U012257	1,297	-	29.99	3720.36	7.56	3.83E-03	-

Unigene ID	Length	Description	FPKM		Log_2FC	P-value	Gene ontology
			NW	AD			
U017647	734	-	29.86	3904.8	7.56	4.60E-04	-
U064367	864	-	0.79	102.24	7.58	7.66E-05	-
U057529	1,528	Probable disease resistance protein At1g61180 [Source:SWISS;ACC:Q940K0]	0.36	47.14	7.65	7.05E-05	GO:0005524, GO:0006952, GO:0043531
U080025	583	-	1.82	274.27	7.72	3.73E-05	-
U046731	830	-	0.17	25	7.77	2.79E-03	-
U003323	1,145	(+)-pulegone reductase [Source:SWISS;ACC:Q6WAU0]	0.11	15.5	7.78	2.79E-03	GO:0005737, GO:0008270, GO:0016114, GO:0042214, GO:0052579, GO:0052580, GO:0070402
U059946	1,865	Geraniol 8-hydroxylase [Source:SWISS;ACC:Q8VWZ7]	1.7	242.44	7.79	1.93E-05	GO:0005506, GO:0005789, GO:0016021, GO:0016099, GO:0016709, GO:0020037
U022500	844	-	0.33	53.06	7.9	2.97E-04	-
U017648	730	-	20.05	3352.05	7.92	1.98E-04	-
U019322	581	Probable non-specific lipid-transfer protein AKCS9 [Source:SWISS;ACC:Q43681]	9.72	1705.88	7.94	1.95E-05	GO:0006810, GO:0008289

Unigene ID	Length	Description	FPKM		Log_2FC	P-value	Gene ontology
			NW	AD			
U012135	1,116	GDSL esterase/lipase At5g55050 [Source:SWISS;ACC:Q9FIA1]	0.11	19.28	8.04	1.46E-03	GO:0005576, GO:0016042, GO:0016298
U029636	731	-	0.2	37.61	8.06	1.40E-03	-
U030041	1,426	Replication protein A 14 kDa subunit B [Source:SWISS;ACC:Q6NLG7]	0.13	22.93	8.06	4.25E-04	GO:0003677, GO:0005634, GO:0006260, GO:0006281, GO:0006310
U008946	766	-	1.14	211.23	8.08	1.56E-05	-
U023147	709	-	0.21	40.76	8.09	1.28E-03	-
U002180	825	-	0.17	31.64	8.1	1.25E-03	-
U082888	577	-	1.24	243.92	8.11	2.99E-05	-
U047288	611	-	0.56	111.23	8.14	1.52E-04	-
U019020	558	-	1.32	271.59	8.16	2.59E-05	-
U031040	788	Protein TIFY 10A [Source:SWISS;ACC:Q9LMA8]	0.4	78.62	8.17	9.64E-05	GO:0005634, GO:0006351, GO:0006355, GO:0009555, GO:0009753, GO:0009867, GO:0009908, GO:0042742
U031336	685	-	0.23	46.09	8.18	1.02E-03	-
U000117	805	Mannose/glucose-specific lectin [Source:SWISS;ACC:P83304]	1.58	336.57	8.28	6.66E-06	GO:0000771, GO:0005536, GO:0005537

Unigene ID	Length	Description	FPKM		Log_2FC	P-value	Gene ontology
			NW	AD			
U015407	982	Bidirectional sugar transporter SWEET7b [Source:SWISS;ACC:Q0J349]	0.13	28.01	8.31	7.26E-04	GO:0005887, GO:0034219, GO:0051119
U017583	651	-	0.25	55.16	8.31	7.40E-04	-
U032925	1,248	-	0.19	42.27	8.39	7.57E-05	-
U022674	2,005	-	0.64	144.37	8.44	4.99E-06	-
U019858	763	Polyubiquitin 11 [Source:SWISS;ACC:P0CH33]	0.57	138.92	8.46	2.19E-05	GO:0005634, GO:0005737, GO:0006511
U045981	808	Endoglucanase [Source:SWISS;ACC:P22503]	0.17	44.51	8.54	3.94E-04	GO:0008810, GO:0009835, GO:0030245
U035032	1,403	Probable dolichyl-diphosphooligosaccharide--protein glycosyltransferase subunit 3B [Source:SWISS;ACC:Q9SYB5]	0.28	67.98	8.55	1.12E-05	GO:0005783, GO:0005886, GO:0006487, GO:0008250, GO:0009507, GO:0016021
U014753	2,462	Zinc finger CCCH domain-containing protein 19 [Source:SWISS;ACC:Q9SIV5]	0.05	11.89	8.57	2.12E-04	GO:0003677, GO:0005634, GO:0006352, GO:0008270, GO:0010964, GO:0016570, GO:0031047, GO:0032776, GO:0042393
U043800	1,078	-	0.12	31.59	8.68	2.71E-04	-
U009822	677	-	0.23	79.7	8.94	1.31E-04	-

Unigene ID	Length	Description	FPKM		Log_2FC	P-value	Gene ontology
			NW	AD			
U002745	832	Agglutinin [Source:SWISS;ACC:P82859]	2.51	860.89	8.98	3.61E-06	GO:0005537, GO:0008061, GO:0051781
U009402	1,391	-	0.08	28.58	9.03	1.01E-04	-
U022677	1,687	-	0.26	89.51	9.03	3.06E-06	-
U000155	579	-	1.83	716.77	9.1	1.91E-06	-
U008729	628	Non-specific lipid-transfer protein [Source:SWISS;ACC:Q39950]	0.26	119.36	9.32	4.43E-05	GO:0006869, GO:0008289
U013787	558	-	0.99	519.24	9.51	1.61E-06	-
U014343	518	Cysteine proteinase inhibitor 5 [Source:SWISS;ACC:Q41916]	1.16	611.84	9.51	1.60E-06	GO:0004869, GO:0005576, GO:0005618, GO:0006952
U013786	1,222	GTP-binding nuclear protein Ran2 [Source:SWISS;ACC:P38547]	0.2	94.95	9.52	3.46E-06	GO:0003924, GO:0005525, GO:0005634, GO:0006886, GO:0006913, GO:0007264
U036205	1,261	F-box/kelch-repeat protein At3g06240 [Source:SWISS;ACC:Q8GXC7]	0.19	97.93	9.6	2.64E-06	-
U040561	6,830	-	0.01	7.43	9.69	1.51E-05	-
U052844	1,601	Luminal-binding protein 5 [Source:SWISS;ACC:Q03685]	0.07	44.47	9.93	7.70E-06	GO:0005524, GO:0005788

Unigene ID	Length	Description	FPKM		Log_2FC	P-value	Gene ontology
			NW	AD			
U017430	1,717	Cytochrome P450 71A4 [Source:SWISS;ACC:P37117]	0.06	43.51	10	5.92E-06	GO:0004497, GO:0005506, GO:0016021, GO:0016705, GO:0020037
U002822	714	Protein RALF-like 24 [Source:SWISS;ACC:Q9LK37]	0.42	317	10.1	9.13E-07	GO:0004871, GO:0005179, GO:0007267, GO:0019722, GO:0048046
U004468	1,537	Uncharacterized mitochondrial protein AtMg00300 [Source:SWISS;ACC:P93293]	0.07	57.17	10.2	3.43E-06	GO:0005739
U021595	7,313	-	0.04	29.08	10.2	4.56E-07	-
U024128	602	Probable non-specific lipid-transfer protein AKCS9 [Source:SWISS;ACC:Q43681]	0.13	105.61	10.2	3.37E-05	GO:0006810, GO:0008289
U081210	612	-	0.28	262.51	10.4	2.18E-06	-
U085276	1,160	-	0.38	329.45	10.4	4.12E-07	-
U021962	3,008	-	0.03	39.03	10.8	7.33E-07	-
U024662	636	-	0.04	53.65	10.8	3.58E-04	-
U028558	4,699	-	0.04	54.96	11	1.71E-07	-
U016597	3,053	-	0.03	45.92	11.1	4.00E-07	-

Unigene ID	Length	Description	FPKM		Log_2FC	P-value	Gene ontology
			NW	AD			
U000795	1,018	ABC transporter E family member 2 [Source:SWISS;ACC:Q8LPJ4]	0.13	196.42	11.2	3.01E-07	GO:0005215, GO:0005524, GO:0016020, GO:0016887, GO:0046872, GO:0051539
U035373	5,485	-	0.02	26.65	11.2	2.99E-07	-
U085275	1,141	-	0.26	467.69	11.4	9.34E-08	-
U007400	748	-	0.39	851.3	11.6	9.68E-08	-
U048305	9,157	-	0.01	20.16	11.6	1.45E-07	-
U019851	2,261	Probable metal-nicotianamine transporter YSL7 [Source:SWISS;ACC:Q9SHY2]	0.01	11.87	11.7	1.09E-04	GO:0005886, GO:0016021, GO:0055085
U036202	858	-	0.16	373.93	11.8	1.07E-07	-
U037701	14,481	-	0.01	29.91	11.8	8.86E-08	-
U030773	523	Pentatricopeptide repeat-containing protein At1g29710, mitochondrial [Source:SWISS;ACC:Q9C6G2]	0.43	1282.78	12	5.95E-08	GO:0005739
U027761	986	-	0.13	399.76	12.2	6.23E-08	-
U056899	1,343	-	0.4	1238.15	12.2	9.37E-07	-

Unigene ID	Length	Description	FPKM		Log_2FC	P-value	Gene ontology
			NW	AD			
U007413	632	-	0	27.23	13.8	4.75E-03	-
U029691	811	-	0	17.36	13.8	4.93E-03	-
U045822	1,890	-	0	5.49	13.8	4.75E-03	-
U057354	529	Nucleosome assembly protein 1 [Source:SWISS;ACC:Q70Z18]	0	38.86	13.8	4.93E-03	GO:0005634, GO:0005737, GO:0006334
U010167	604	-	0	31.72	13.9	3.79E-03	-
U019602	1,264	-	0	9.65	13.9	3.93E-03	-
U023801	781	-	0	19.67	13.9	3.93E-03	-
U025485	1,409	Putative receptor protein kinase ZmPK1 [Source:SWISS;ACC:P17801]	0	8.21	13.9	4.24E-03	GO:0004674, GO:0005524, GO:0016021, GO:0048544
U026284	591	-	0	33.13	13.9	3.79E-03	-
U027731	886	Polyubiquitin [Source:SWISS;ACC:P0CG73]	0	15.38	13.9	4.57E-03	GO:0005634, GO:0005737
U028668	688	-	0	23.5	13.9	4.57E-03	-
U031837	861	Transactivator/viroplasmin protein [Source:SWISS;ACC:P15628]	0	16.78	13.9	3.93E-03	GO:0006417, GO:0030430

Unigene ID	Length	Description	FPKM		Log_2FC	P-value	Gene ontology
			NW	AD			
U034831	980	-	0	13.15	13.9	4.57E-03	-
U038252	925	APO protein 1, chloroplastic [Source:SWISS;ACC:Q9XIR4]	0	15.2	13.9	3.79E-03	GO:0003723, GO:0009507
U048636	1,125	Putative ribonuclease H protein At1g65750 [Source:SWISS;ACC:P0C2F6]	0	11.46	13.9	3.79E-03	GO:0003676, GO:0004523, GO:0046872
U060314	524	Cell number regulator 3 [Source:SWISS;ACC:D9HP19]	0	40.57	13.9	4.57E-03	GO:0016021
U061467	528	-	0	41.19	13.9	4.08E-03	-
U076948	566	-	0	34.35	13.9	4.57E-03	-
U080443	2,191	Adenylate kinase 9 [Source:SWISS;ACC:Q5TCS8]	0	4.66	13.9	4.57E-03	GO:0004550, GO:0005524, GO:0005634, GO:0005730, GO:0005737, GO:0006174, GO:0006186, GO:0006756, GO:0006757, GO:0031965, GO:0050145, GO:0061508, GO:0061565, GO:0061566, GO:0061567, GO:0061568, GO:0061569, GO:0061570, GO:0061571
U083752	503	-	0	46.65	13.9	3.93E-03	-
U084409	569	-	0	34.54	13.9	4.24E-03	-
U084859	899	Unconventional myosin-X [Source:SWISS;ACC:P79114]	0	15.85	13.9	3.79E-03	GO:0001726, GO:0005524, GO:0005547, GO:0005829, GO:0005938, GO:0006200,

Unigene ID	Length	Description	FPKM		Log_2FC	P-value	Gene ontology
			NW	AD			
							GO:0008152, GO:0008360, GO:0016459, GO:0030027, GO:0030705, GO:0030898, GO:0031527, GO:0032433, GO:0051015, GO:0051489, GO:0060002
U089563	974	-	0	13.73	13.9	4.08E-03	-
U095550	1,207	-	0	9.99	13.9	4.24E-03	-
U018971	597	Bet1-like SNARE 1-1 [Source:SWISS;ACC:Q9M2J9]	0	33.66	14	3.28E-03	GO:0000139, GO:0005484, GO:0005634, GO:0005789, GO:0005886, GO:0015031, GO:0016021, GO:0016192
U020262	506	-	0	49	14	3.05E-03	-
U020862	967	Nudix hydrolase 22, chloroplastic [Source:SWISS;ACC:O22951]	0	14.57	14	3.40E-03	GO:0009507, GO:0016787, GO:0046872
U024377	960	Purple acid phosphatase 8 [Source:SWISS;ACC:Q8VYZ2]	0	14.79	14	3.40E-03	GO:0003993, GO:0005576, GO:0016311, GO:0046872
U036811	951	-	0	15.65	14	2.94E-03	-
U040179	605	-	0	32.59	14	3.40E-03	-
U052740	980	RING finger and CHY zinc finger domain-containing protein 1 [Source:SWISS;ACC:Q96PM5]	0	14.06	14	3.65E-03	GO:0000151, GO:0002039, GO:0004842, GO:0005634, GO:0005737, GO:0008270, GO:0016567, GO:0016607, GO:0016874, GO:0031398, GO:0032436, GO:0042787, GO:0042803, GO:0051865

Unigene ID	Length	Description	FPKM		Log_2FC	P-value	Gene ontology
			NW	AD			
U058878	1,178	-	0	11.26	14	3.16E-03	-
U089053	582	-	0	36.3	14	3.05E-03	-
U093099	896	-	0	16.95	14	3.05E-03	-
U095720	751	Probably inactive leucine-rich repeat receptor-like protein kinase At3g28040 [Source:SWISS;ACC:Q9LRT1]	0	22.2	14	3.28E-03	GO:0004672, GO:0005524, GO:0016021
U000317	1,386	-	0	9.4	14.1	2.74E-03	-
U011817	2,463	-	0	4.78	14.1	2.56E-03	-
U016598	1,335	-	0	10.46	14.1	2.23E-03	-
U022226	687	-	0	28.17	14.1	2.39E-03	-
U035081	869	Transcription factor ORG2 [Source:SWISS;ACC:Q9M1K1]	0	19	14.1	2.39E-03	GO:0003677, GO:0003700, GO:0005634, GO:0006351, GO:0006355, GO:0010106, GO:0055072
U035399	941	ATP-dependent Clp protease proteolytic subunit 5, chloroplastic [Source:SWISS;ACC:Q9S834]	0	16.42	14.1	2.56E-03	GO:0004252, GO:0009507, GO:0009532, GO:0009534, GO:0009570, GO:0009579, GO:0009840, GO:0009941
U045842	1,034	-	0	14.81	14.1	2.23E-03	-
U060833	521	-	0	50.03	14.1	2.23E-03	-

Unigene ID	Length	Description	FPKM		Log_2FC	P-value	Gene ontology
			NW	AD			
U070695	563	ADP-ribosylation factor GTPase-activating protein AGD3 [Source:SWISS;ACC:Q5W7F2]	0	41.9	14.1	2.31E-03	GO:0005829, GO:0005886, GO:0006897, GO:0008060, GO:0008270, GO:0009733, GO:0009965, GO:0010051, GO:0010087, GO:0030140, GO:0032312, GO:0035091, GO:0043547
U077558	900	-	0	17.46	14.1	2.65E-03	-
U080814	1,620	-	0	8.09	14.1	2.31E-03	-
U091553	1,530	-	0	8.7	14.1	2.31E-03	-
U098113	859	-	0	18.99	14.1	2.56E-03	-
U023800	677	-	0	30.01	14.2	2.09E-03	-
U025371	793	AP-2 complex subunit sigma [Source:SWISS;ACC:Q84WL9]	0	23.9	14.2	1.72E-03	GO:0006897, GO:0008565, GO:0030122
U026924	842	-	0	21.09	14.2	1.95E-03	-
U029820	1,733	Cactin [Source:SWISS;ACC:F4I2J8]	0	7.58	14.2	2.16E-03	GO:0005681, GO:0006397, GO:0008380, GO:0016607
U030433	844	-	0	21.29	14.2	1.83E-03	-
U031849	1,273	F-box/kelch-repeat protein At3g18720 [Source:SWISS;ACC:Q9LSA5]	0	11.4	14.2	2.02E-03	-

Unigene ID	Length	Description	FPKM		Log_2FC	P-value	Gene ontology
			NW	AD			
U044783	2,201	Histidine kinase 2 [Source:SWISS;ACC:Q9C5U2]	0	6.04	14.2	1.72E-03	GO:0000155, GO:0004673, GO:0005034, GO:0005789, GO:0005886, GO:0006970, GO:0007275, GO:0009414, GO:0009636, GO:0009651, GO:0009736, GO:0009737, GO:0009884, GO:0009909, GO:0010029, GO:0010087, GO:0010271, GO:0016021, GO:0018106, GO:0019900, GO:0034757, GO:0042802, GO:0043424, GO:0048509, GO:0048831, GO:0070417, GO:0071215, GO:0080117
U061786	837	-	0	21.58	14.2	1.83E-03	-
U061890	588	-	0	40.38	14.2	1.89E-03	-
U086415	535	-	0	48.02	14.2	2.09E-03	-
U011966	598	-	0	41.36	14.3	1.51E-03	-
U012737	2,905	-	0	4.59	14.3	1.47E-03	-
U017581	661	-	0	33.26	14.3	1.66E-03	-
U029568	974	26S proteasome non-ATPase regulatory subunit 4 homolog [Source:SWISS;ACC:P55034]	0	17.66	14.3	1.61E-03	GO:0000502, GO:0001653, GO:0005634, GO:0005829, GO:0006974, GO:0007165, GO:0008540, GO:0009408, GO:0009555, GO:0009651, GO:0009733, GO:0009735, GO:0009737, GO:0009744, GO:0010029, GO:0010150, GO:0016020, GO:0030163, GO:0031593, GO:0043161, GO:0043248, GO:0048366, GO:0048455, GO:0048528, GO:0048767, GO:0051788

Unigene ID	Length	Description	FPKM		Log_2FC	P-value	Gene ontology
			NW	AD			
U033899	1,016	-	0	17	14.3	1.47E-03	-
U041669	672	-	0	33.06	14.3	1.51E-03	-
U049134	574	Non-specific lipid-transfer protein [Source:SWISS;ACC:P27631]	0	44.15	14.3	1.61E-03	GO:0006869, GO:0008289
U053456	1,619	Probable mitochondrial chaperone bcs1 [Source:SWISS;ACC:Q9P6Q3]	0	9.3	14.3	1.38E-03	GO:0005524, GO:0005739, GO:0005743, GO:0006200, GO:0016021, GO:0016226, GO:0034551, GO:0042623, GO:0043623
U096455	594	-	0	43.01	14.3	1.38E-03	-
U099152	630	-	0	38.54	14.3	1.34E-03	-
U002037	534	-	0	55.83	14.4	1.19E-03	-
U014544	1,610	G-type lectin S-receptor-like serine/threonine-protein kinase At1g34300 [Source:SWISS;ACC:Q9XID3]	0	9.57	14.4	1.26E-03	GO:0004674, GO:0005516, GO:0005524, GO:0005886, GO:0016021, GO:0030246, GO:0048544
U015311	847	-	0	24.08	14.4	1.13E-03	-
U022765	1,424	Wall-associated receptor kinase-like 9 [Source:SWISS;ACC:Q9C9L5]	0	11.06	14.4	1.30E-03	GO:0004674, GO:0005509, GO:0005524, GO:0016021, GO:0030247
U029170	871	-	0	23.52	14.4	1.03E-03	-

Unigene ID	Length	Description	FPKM		Log_2FC	P-value	Gene ontology
			NW	AD			
U038570	912	Putative receptor-like protein kinase At5g39000 [Source:SWISS;ACC:Q9FID8]	0	20.78	14.4	1.26E-03	GO:0004674, GO:0005524, GO:0016021
U040558	1,706	-	0	9.03	14.4	1.19E-03	-
U077901	520	-	0	58.6	14.4	1.23E-03	-
U006244	1,241	Putative ribonuclease H protein At1g65750 [Source:SWISS;ACC:P0C2F6]	0	14.2	14.5	1.01E-03	GO:0003676, GO:0004523, GO:0046872
U010190	2,536	-	0	6.08	14.5	9.03E-04	-
U014741	992	-	0	20.01	14.5	9.03E-04	-
U016571	919	-	0	22.91	14.5	8.35E-04	-
U016944	586	-	0	49.09	14.5	9.28E-04	-
U018202	602	-	0	48.34	14.5	7.92E-04	-
U019351	1,233	-	0	14.64	14.5	9.28E-04	-
U034030	602	-	0	46.6	14.5	9.03E-04	-
U055694	1,647	-	0	10.24	14.5	8.80E-04	-

Unigene ID	Length	Description	FPKM		Log_2FC	P-value	Gene ontology
			NW	AD			
U084616	1,540	-	0	11.34	14.5	8.13E-04	-
U002823	2,858	-	0	5.91	14.6	6.03E-04	-
U009339	2,724	-	0	5.88	14.6	7.53E-04	-
U010753	4,602	-	0	3.39	14.6	6.81E-04	-
U014953	1,032	Pentatricopeptide repeat-containing protein At1g63150 [Source:SWISS;ACC:Q9CAM8]	0	20.35	14.6	6.81E-04	-
U031368	792	-	0	30.12	14.6	7.34E-04	-
U040560	3,289	-	0	4.97	14.6	6.32E-04	-
U052990	1,600	Probable inactive receptor kinase At5g10020 [Source:SWISS;ACC:Q0WR59]	0	11.73	14.6	6.03E-04	GO:0004672, GO:0005524, GO:0005829, GO:0005886, GO:0016021
U061803	500	-	0	74.59	14.6	6.98E-04	-
U007659	1,658	-	0	11.48	14.7	5.48E-04	-
U011380	890	-	0	28.05	14.7	4.66E-04	-
U015169	753	Fatty-acid-binding protein 1 [Source:SWISS;ACC:Q9M1X2]	0	35.3	14.7	5.48E-04	GO:0005504, GO:0005739, GO:0006631, GO:0009507, GO:0009536, GO:0009570, GO:0009813, GO:0009941, GO:0045430

Unigene ID	Length	Description	FPKM		Log_2FC	P-value	Gene ontology
			NW	AD			
U029381	888	Membrane-anchored ubiquitin-fold protein 4 [Source:SWISS;ACC:Q9LSD8]	0	27.1	14.7	5.35E-04	GO:0005886
U029383	888	Membrane-anchored ubiquitin-fold protein 4 [Source:SWISS;ACC:Q9LSD8]	0	27.1	14.7	5.35E-04	GO:0005886
U040564	2,937	-	0	5.91	14.7	5.35E-04	-
U002420	760	-	0	37.97	14.8	3.90E-04	-
U005679	553	-	0	69.82	14.8	3.82E-04	-
U010617	1,023	Putative F-box/LRR-repeat protein 23 [Source:SWISS;ACC:Q9S9V9]	0	24.16	14.8	3.73E-04	-
U010731	727	-	0	40.63	14.8	4.07E-04	-
U010822	3,536	-	0	5.28	14.8	3.73E-04	-
U015824	964	-	0	26.02	14.8	3.90E-04	-
U015835	526	-	0	78.71	14.8	3.65E-04	-
U015958	1,313	3-oxoacyl-[acyl-carrier-protein] reductase, chloroplastic [Source:SWISS;ACC:P28643]	0	17.03	14.8	3.82E-04	GO:0004316, GO:0006633, GO:0009507, GO:0051287

Unigene ID	Length	Description	FPKM		Log_2FC	P-value	Gene ontology
			NW	AD			
U017511	559	-	0	66.83	14.8	4.17E-04	-
U017582	610	-	0	55.79	14.8	4.17E-04	-
U021470	1,186	Acyl-CoA--sterol O-acyltransferase 1 [Source:SWISS;ACC:Q9SV07]	0	18.6	14.8	4.55E-04	GO:0016021, GO:0016127, GO:0016746, GO:0034434
U031059	826	-	0	33.26	14.8	3.82E-04	-
U052350	2,524	Arginine--tRNA ligase {ECO:0000255|HAMAP-Rule:MF_00123} [Source:SWISS;ACC:Q82E68]	0	7.5	14.8	4.17E-04	GO:0004814, GO:0005524, GO:0005737, GO:0006420
U087382	636	-	0	51.95	14.8	3.99E-04	-
U017584	550	-	0	74.18	14.9	3.15E-04	-
U002273	639	-	0	60.36	15	2.14E-04	-
U013642	522	-	0	89.4	15	2.36E-04	-
U014752	2,477	Zinc finger CCCH domain-containing protein 19 [Source:SWISS;ACC:Q9SIV5]	0	8.64	15	2.56E-04	GO:0003677, GO:0005634, GO:0006352, GO:0008270, GO:0010964, GO:0016570, GO:0031047, GO:0032776, GO:0042393
U017291	4,310	-	0	4.71	15	2.51E-04	-

Unigene ID	Length	Description	FPKM		Log_2FC	P-value	Gene ontology
			NW	AD			
U035832	1,717	Uncharacterized acetyltransferase At3g50280 [Source:SWISS;ACC:Q9SND9]	0	13.35	15	2.56E-04	GO:0016747
U078454	661	-	0	54.5	15	2.51E-04	-
U082491	816	-	0	37.55	15	2.56E-04	-
U000060	854	-	0	37.71	15.1	1.87E-04	-
U010762	1,663	-	0	14.89	15.1	1.95E-04	-
U017814	668	-	0	57	15.1	1.95E-04	-
U037715	1,399	-	0	19.15	15.1	1.70E-04	-
U059276	526	Flavin-containing monooxygenase FMO GS-OX-like 3 [Source:SWISS;ACC:Q9SXD5]	0	93.14	15.1	1.87E-04	GO:0004499, GO:0050660, GO:0050661
U061030	1,250	-	0	22.47	15.1	1.61E-04	-
U085078	505	-	0	106.15	15.1	1.61E-04	-
U090022	831	-	0	41.06	15.1	1.58E-04	-
U008503	514	-	0	104.55	15.2	1.45E-04	-

Unigene ID	Length	Description	FPKM		Log_2FC	P-value	Gene ontology
			NW	AD			
U035098	592	-	0	81.08	15.2	1.19E-04	-
U050922	1,918	NEDD8-activating enzyme E1 regulatory subunit {ECO:0000305} [Source:SWISS;ACC:P42744]	0	13.34	15.2	1.50E-04	GO:0000166, GO:0003824, GO:0005634, GO:0005829, GO:0006281, GO:0007131, GO:0009414, GO:0009734, GO:0009735, GO:0009965, GO:0010252, GO:0016567, GO:0045116
U000654	2,327	-	0	11.39	15.3	1.13E-04	-
U019372	2,858	-	0	9.13	15.3	1.07E-04	-
U040121	1,365	Cationic amino acid transporter 9, chloroplastic [Source:SWISS;ACC:Q9C5D6]	0	22.31	15.3	1.04E-04	GO:0005774, GO:0015171, GO:0016021, GO:0031969
U051888	728	-	0	57.75	15.3	9.70E-05	-
U007660	2,728	-	0	10.48	15.4	7.49E-05	-
U014568	983	-	0	37.96	15.4	7.49E-05	-
U019134	4,591	-	0	5.75	15.4	8.37E-05	-
U026362	1,099	-	0	33.07	15.4	6.82E-05	-
U079954	774	-	0	55.11	15.4	7.61E-05	-
U022834	1,067	-	0	36.09	15.5	5.60E-05	-

Unigene ID	Length	Description	FPKM		Log_2FC	P-value	Gene ontology
			NW	AD			
U047287	545	-	0	112.87	15.5	6.12E-05	-
U013183	2,473	-	0	13.61	15.6	3.97E-05	-
U019371	2,608	-	0	12.96	15.6	3.76E-05	-
U102955	544	-	0	131.6	15.7	3.24E-05	-
U011899	4,371	-	0	8.36	15.8	2.14E-05	-
U025260	950	-	0	54.19	15.8	2.04E-05	-
U001046	808	-	0	73.53	15.9	1.63E-05	-
U040557	5,011	-	0	7.63	15.9	1.69E-05	-
U019170	5,234	-	0	7.92	16	1.17E-05	-
U051036	2,297	DNA topoisomerase 2 [Source:SWISS;ACC:P30182]	0	18.83	16	1.41E-05	GO:0000712, GO:0000795, GO:0000819, GO:0003677, GO:0003916, GO:0003918, GO:0005524, GO:0005634, GO:0006200, GO:0006261, GO:0006265, GO:0006312, GO:0008094, GO:0009330, GO:0044774, GO:0046872
U056819	4,211	-	0	10.12	16	1.11E-05	-
U016865	1,798	Histone-lysine N-methyltransferase ATXR2 [Source:SWISS;ACC:Q5PP37]	0	27.82	16.1	9.19E-06	GO:0005634, GO:0018024, GO:0046872

Unigene ID	Length	Description	FPKM		Log_2FC	P-value	Gene ontology
			NW	AD			
U035806	1,214	Albumin-2 [Source:SWISS;ACC:P08688]	0	47.26	16.1	8.16E-06	GO:0005509, GO:0005829, GO:0045735
U064454	503	-	0	213.89	16.1	8.49E-06	-
U086794	550	-	0	166.61	16.1	1.06E-05	-
U007933	1,175	-	0	51.66	16.2	6.76E-06	-
U093739	905	-	0	73.14	16.2	7.71E-06	-
U023862	3,294	-	0	16.01	16.3	4.90E-06	-
U033385	1,205	Rop guanine nucleotide exchange factor 1 [Source:SWISS;ACC:Q93ZY2]	0	53.7	16.3	4.90E-06	GO:0005089, GO:0005829, GO:0009860, GO:0016324, GO:0032321, GO:0080092
U012313	618	-	0	161.9	16.4	4.29E-06	-
U034170	996	-	0	74.61	16.4	3.79E-06	-
U031348	667	-	0	168.96	16.6	1.93E-06	-
U050100	2,537	Arginine--tRNA ligase {ECO:0000255|HAMAP-Rule:MF_00123} [Source:SWISS;ACC:B7KCT7]	0	26.48	16.6	2.01E-06	GO:0004814, GO:0005524, GO:0005737, GO:0006420
U092211	643	-	0	182.71	16.6	1.86E-06	-

Unigene ID	Length	Description	FPKM		Log_2FC	P-value	Gene ontology
			NW	AD			
U007380	5,436	-	0	12.33	16.7	1.49E-06	-
U019844	5,236	-	0	12.68	16.7	1.57E-06	-
U024325	7,193	-	0	8.96	16.7	1.65E-06	-
U063181	652	-	0	181.04	16.7	1.73E-06	-
U000069	714	-	0	172.45	16.8	1.06E-06	-
U015178	792	-	0	141.38	16.8	1.16E-06	-
U019192	1,069	-	0	89.39	16.8	1.16E-06	-
U036209	1,059	F-box/kelch-repeat protein At3g06240 [Source:SWISS;ACC:Q8GXC7]	0	90.08	16.8	1.19E-06	-
U102440	713	-	0	181.85	16.9	8.67E-07	-
U059347	1,196	-	0	97.33	17.2	4.42E-07	-
U102706	731	-	0	216.23	17.2	3.69E-07	-
U051034	3,020	DNA topoisomerase 2 [Source:SWISS;ACC:P30182]	0	34.28	17.3	3.24E-07	GO:0000712, GO:0000795, GO:0000819, GO:0003677, GO:0003916, GO:0003918, GO:0005524, GO:0005634, GO:0006200, GO:0006261, GO:0006265, GO:0006312, GO:0008094, GO:0009330, GO:0044774, GO:0046872

Unigene ID	Length	Description	FPKM		Log_2FC	P-value	Gene ontology
			NW	AD			
U100830	813	-	0	323.74	18.1	5.29E-08	-
U013696	1,012	-	0	258.62	18.2	3.92E-08	-
U046194	620	-	0	610.01	18.3	3.56E-08	-
U002510	1,103	-	0	381.27	19	1.48E-08	-

Supplementary Table S4. Three highest-ranked functional clusters of up-regulated genes determined by DAVID gene functional classification tool

Category or term	Functional annotation	No. DEGs	Fold enrichment
Annotation cluster 1 (enrichment score: 1.34)			
GO:0009055	Electron carrier activity	6	3.43
IPR017972	Cytochrome P450, conserved site	11	7.05
IPR002401	Cytochrome P450, E-class, group I	11	6.96
IPR017973	Cytochrome P450, C-terminal region	12	6.79
IPR001128	Cytochrome P450	6	6.63
SP_PIR_KEYWORDS	Iron	3	3.11
PIRSF000045	Cytochrome P450 CYP2D6	5	11.21
SP_PIR_KEYWORDS	Monooxygenase	5	5.74
GO:0005506	Iron ion binding	6	2.63
SP_PIR_KEYWORDS	Heme	6	4.01
GO:0055114	Oxidation reduction	6	1.91
GO:0020037	Heme binding	11	3.62
GO:0046906	Tetrapyrrole binding	11	3.17
SP_PIR_KEYWORDS	Metalloprotein	12	4.07
SP_PIR_KEYWORDS	Oxidoreductase	6	1.47
Annotation cluster 2 (enrichment score: 0.76)			
GO:0017076	Purine nucleotide binding	19	1.38
GO:0032555	Purine ribonucleotide binding	18	1.37
GO:0032553	Ribonucleotide binding	18	1.37
SP_PIR_KEYWORDS	Nucleotide-binding	16	1.41
GO:0030554	Adenyl nucleotide binding	17	1.35
GO:0001883	Purine nucleoside binding	17	1.35
GO:0001882	Nucleoside binding	17	1.34
GO:0005524	ATP binding	16	1.36
GO:0032559	Adenyl ribonucleotide binding	16	1.34
SP_PIR_KEYWORDS	ATP-binding	14	1.40
GO:0000166	Nucleotide binding	20	1.26
UP_SEQ_FEATURE	Nucleotide phosphate-binding region:ATP	9	1.45
Annotation cluster 3 (enrichment score: 0.75)			
UP_SEQ_FEATURE	Repeat:LRR 5	4	3.71
UP_SEQ_FEATURE	Repeat:LRR 4	4	3.21
UP_SEQ_FEATURE	Repeat:LRR 3	4	2.86
UP_SEQ_FEATURE	Repeat:LRR 2	4	2.66
UP_SEQ_FEATURE	Repeat:LRR 1	3	2.66
UP_SEQ_FEATURE	Repeat:LRR 6	4	3.40
SP_PIR_KEYWORDS	Leucine-rich repeat	3	2.24
IPR001611	Leucine-rich repeat	4	2.39

Supplementary Table S5. Three highest-ranked functional clusters of down-regulated genes determined using the DAVID gene functional classification tool

Category	Term	No. DEGs	Fold enrichment
Annotation cluster 1 (enrichment score: 1.06)			
GO:0009699	Phenylpropanoid biosynthetic process	4	5.82
GO:0019438	Aromatic compound biosynthetic process	5	4.02
GO:0009698	Phenylpropanoid metabolic process	4	4.25
GO:0042398	Cellular amino acid derivative biosynthetic process	4	3.73
GO:0006575	Cellular amino acid derivative metabolic process	4	2.35
GO:0019748	Secondary metabolic process	4	1.94
Annotation cluster 2 (enrichment score: 0.85)			
SM00256	FBOX	7	2.64
UP_SEQ_FEATURE	Domain:F-box	7	2.43
IPR001810	Cyclin-like F-box	7	2.26
Annotation cluster 3 (enrichment score: 0.83)			
GO:0009735	Response to cytokinin stimulus	6	9.10
GO:0009725	Response to hormone stimulus	11	2.08
GO:0009719	Response to endogenous stimulus	11	1.95
GO:0010033	Response to organic substance	12	1.74
GO:0009733	Response to auxin stimulus	6	2.93
GO:0009734	Auxin mediated signaling pathway	3	4.19
GO:0009755	Hormone-mediated signaling	5	2.32
GO:0032870	Cellular response to hormone stimulus	5	2.24
GO:0007242	Intracellular signaling cascade	6	1.49
UP_SEQ_FEATURE	Mutagenesis site	6	1.45
